# Cyclometalated Iridium(III) Complexes as AIE Phosphorescent Probes for Real-Time Monitoring of Mitophagy in Living Cells

**DOI:** 10.1038/srep22039

**Published:** 2016-02-24

**Authors:** Chengzhi Jin, Jiangping Liu, Yu Chen, Ruilin Guan, Cheng Ouyang, Yanjiao Zhu, Liangnian Ji, Hui Chao

**Affiliations:** 1 MOE Key Laboratory of Bioinorganic and Synthetic Chemistry, School of Chemistry and Chemical Engineering, Sun Yat-Sen University, Guangzhou, 510275, China; 2 School of Materials Science and Engineering, Hubei University, Wuhan, 430062, China

## Abstract

Mitophagy, which is a special autophagy that removes damaging mitochondria to maintain sufficient healthy mitochondria, provides an alternative path for addressing dysfunctional mitochondria and avoiding cellular death. In the present study, by coupling the triphenylamine group with 2-phenylimidazo[4,5-*f*][1,10]phenanthroline derivatives, we synthesized five Ir(III) complexes with an AIE property that are expected to fulfill requirements for real-time monitoring of mitophagy. **Ir1-Ir5** were exploited to image mitochondria with a short incubation time by confocal microscopy and inductive coupled plasma–mass spectrometry (ICP-MS). Due to aggregation-induced emission (AIE), **Ir1-Ir5** exhibited excellent photostability compared to MitoTracker Green (MTG). Moreover, **Ir1-Ir5** manifested satisfactory photostability in the mitochondrial physiological pH range. In addition, the uptake mechanism of **Ir1** was investigated using confocal microscopy and flow cytometry analysis. Finally, using both **Ir1** and LysoTracker Green, we were able to achieve real-time monitoring of mitophagy.

As a membrane bound organelle, mitochondria is best known for its critical function involving energy production via oxidative phosphorylation^1^. Although mitochondria have maintained the double membrane character of their ancestors, they have acquired various additional functions within the cell due to evolution, such as energy production, apoptosis regulation, central metabolism, calcium modulation and redox signaling^2^,^3^,^4^. The reactive oxygen species (ROS) generated in the phosphorylation process have the potential to cause mitochondrial dysfunction via damage to mitochondrial proteins, DNA and lipids^5^. Extensive damage to the mitochondria may eventually lead to cell death due to their regulation of apoptosis^6^. Although cell death can benefit an organism, aberrant cell death is detrimental to the organism due to the premature loss of cells, especially when the cells that are lost are post-mitotic cell types, such as neurons^7^. Mitophagy, which is a well-known form of autophagy that removes damaged mitochondria, can maintain a healthy mitochondrial pool and overcome the shortage caused by cellular loss in apoptosis or necrosis^5^,^8^. Microcosmic information of the physiological changes in mitophagy and may predict mitochondrial-related macroscopic diseases, including cardiovascular diseases and neurodegenerative diseases^9^,^10^.

To obtain a high quality image on the subcellular level, many organelle specific agents, such as Hoechest 33342, Nonyl acridine orange and MitoTracker Red, have been commercialized^11^. However, at a dilute concentration, these dyes can be easily photobleached under continuous laser excitation. In addition, the photostability cannot be improved using a higher concentration of most commercial agents for the effect of concentration quenching^12^. Luminogens with aggregation-induced emission (AIE) characteristics exhibit almost no fluorescence when they are molecularly dissolved but become highly emissive in the aggregated state^13^. Strong fluorescence in an aggregate may resolve fluorescence quenching resulting from aggregation, which would promises a long period of observation without large luminescent signal loss^14^,^15^. Thus, in biological areas, agents with AIE characteristics have been used as probes for the sensing of biogenic species or as stains/contrast agents for the imaging of cells^16^,^17^,^18^,^19^,^20^,^21^.

In recent years, due to their large Stokes shifts (more than 100 nm), rapid transmembrane activity (short incubation time and less potential toxicity), long luminescence lifetimes (100 ns to 1 ms), and enhanced photostabilities (less photobleaching), phosphorescent cyclometalated cationic Ir(III) complexes for use as organelle labels and sensors have attracted much attention in the field of bioimaging and labeling^22^,^23^,^24^,^25^. Moreover, altering the structure can conveniently direct the location of these Ir(III) complexes to various subcellular region, such as the nucleus, cytoplasm, lysosome, mitochondria and Golgi apparatus^26^,^27^,^28^,^29^,^30^,^31^,^32^, or result in different optical properties, such as two-photon emission, AIE and near-infrared emission^14^,^33^,^34^. Therefore, to a great extent, the modification of the structure of some existing Ir(III) complexes may result in the production of a new property.

In previous studies, we have found that some Ir(III) complexes possess a high specificity for mitochondria and superior photostability, and can be used for mitochondrial imaging and tracking^14^,^32^,^35^,^36^,^37^. As for mitophagy monitoring, the impaired mitochondria involved in mitophagy would undergo a sophisticated process for a long period of time including not only short-time dynamics change mentioned in our previous work, but also pH fluctuation, violent morphology alteration and membrane potential lost^8^,^38^. Hence, compared to dynamics tracking, probes for mitophagy monitoring are required to manifest higher biocompability, photostability and pH stability to enable long time monitoring in a complicated process. With these requirements mentioned above, we envisaged to combine AIE property with Ir(III) complexes to expect a favorable mitophagy probe. To the best of our knowledge, no AIE iridium(III) probes have been used as a mitochondrial probe for real-time mitophagy monitoring. Therefore, in this study, triphenylamine (TPA) group, a propeller-like structure with electron-donating properties which may bring AIE characteristics^39^,^40^, was introduced to iridium(III) complexes with 1-phenyl-1H-imidazo[4,5-*f*][1,10]phenanthroline derivatives, and the resulting AIE iridium(III) complexes should exhibit good photostability, large Stokes shifts and favorable transmembrane activity. The cellular location of these Ir(III) complexes was determined using confocal microscopy with MitoTracker Green FM (MTG) co-staining and inductively coupled plasma mass spectrometry (ICP-MS). We investigated the cytotoxicity and cellular uptake mechanism of all five Ir(III) complexes (**Ir1–Ir5**) as well as the phosphorescence stability of **Ir1–Ir5** under photobleaching and pH fluctuation. In addition, **Ir1** as well as LysoTracker Green were used for real-time monitoring of the mitophagy process.

## Results and Discussion

### Synthesis and characterization

**Figure 1:**
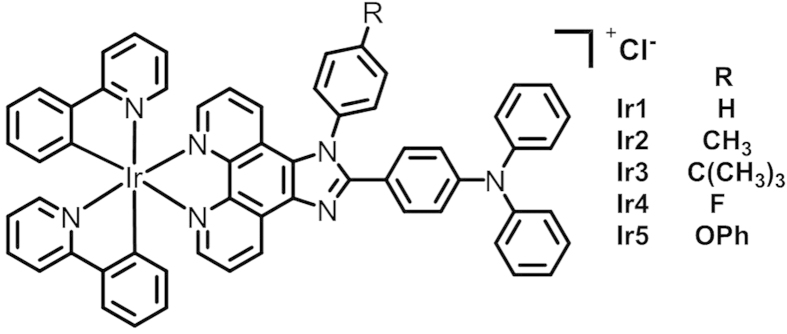
Chemical structures of the Ir1–Ir5 complexes.

A series of N,N-diphenyl-4-(1-phenyl-1H-imidazo[4,5-*f*][1,10]phenanthrolin-2-yl)aniline derivatives (dippa, dtipa, dbipa, dfipa and dpoipa) were synthesized using “one-pot” method. The mixture of 4-(diphenylamino)benzaldehyde, 1,10-phenanthroline-5,6-dione, ammonium chloride and substituted-aniline were refluxed in acetic acid to yield the chosen ligands^35^,^41^. We prepared five Ir(III) complexes (see Fig. 1) by coordinating the corresponding ligands with [Ir(ppy)_2_Cl]_2_ in refluxed CHCl_3_/CH_2_OH (2:1). These complexes were purified by column chromatography and characterized by elemental analysis, ES-MS and ^1^H NMR spectroscopy (Figs S1–S10).

### Absorption and emission spectroscopy

The photophysical data are summarized in Table S1. The UV-Vis absorption spectra of Ir1-Ir5 in the DMSO/PBS solution are shown in Fig. S11. These complexes exhibited weak absorption bands in the region of 330–420 nm and stronger absorption bands in the region of 250–325 nm, both of which can be attributed to the mixed singlet and triplet metal-to-ligand charge-transfers (^1^MLCT and ^3^MLCT) and intra-ligand charge trasfers (C-N ligands)^42^. All five complexes reach maximum emission peaks at approximately 590 nm. These complexes enjoy the advantage of much larger Stokes shifts (around 210 nm) over that of commercial organic cellular dyes, such as LysoTracker Green DND-26 and BIDOPY 493/503 (Stokes shift = 7 nm and 10 nm, respectively)^11^.

### Aggregation-induced emission (AIE) properties

**Figure 2:**
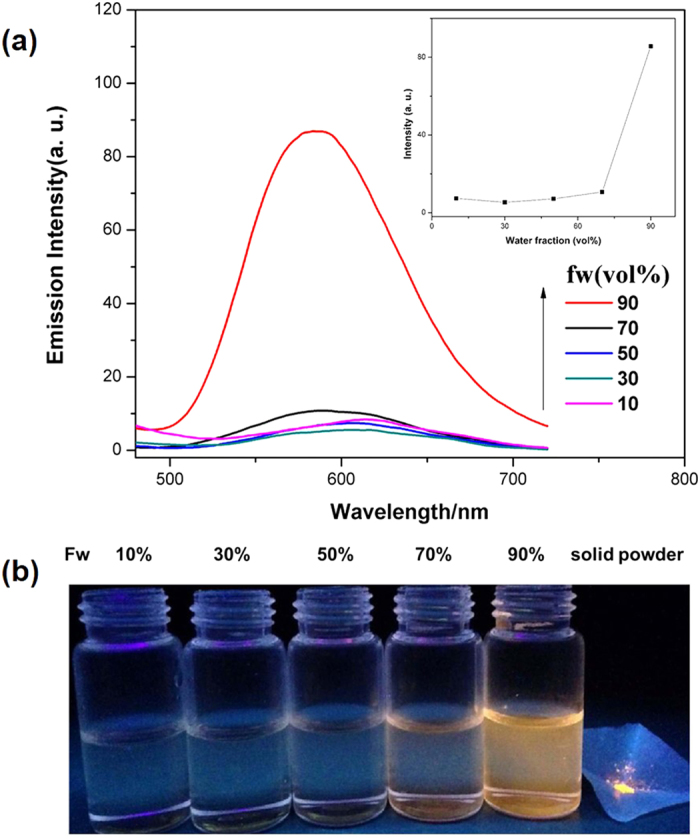
(**a**) Emission spectra of **Ir1** in DMSO–PBS mixtures with different water fractions (fw); (**b**) Images of the room temperature luminescent emissions of **Ir1** solid powder and **Ir1** in DMSO–PBS mixtures with different water fractions (fw).

The obtained complexes (Ir1-Ir5) readily dissolved in many organic solvents, such as chloroform, DMSO, acetonitrile, dichloromethane, ethanol and methanol. However, these complexes exhibited dissatisfactory water solubility. To determine whether these five Ir(III) complexes are AIE active and consider the biological application, the fluorescent behaviors of their diluted mixtures were studied in a mixture of PBS and DMSO with different water fractions. For example, as shown in Fig. 2, when the water content of the mixture was less than 70%, the Ir1 mixture exhibited almost no emission, and the photoluminescence intensity was significantly enhanced when the water content was increased to 90%, which is approximately 20-fold higher than that in low water content solvent. Other Ir(III) complexes exhibit similar properties (Figs S12–S15). The DLS experiments further confirmed the aggregation of Ir1 upon addition of water, and the presence of nanoparticles was observed at 148.4–433.8 nm in DMSO-PBS (9/1, v/v) (Figs S16–S20). These results indicate that Ir1-Ir5 exhibit an AIE effect.

### Cytotoxicity measurements

**Figure 3:**
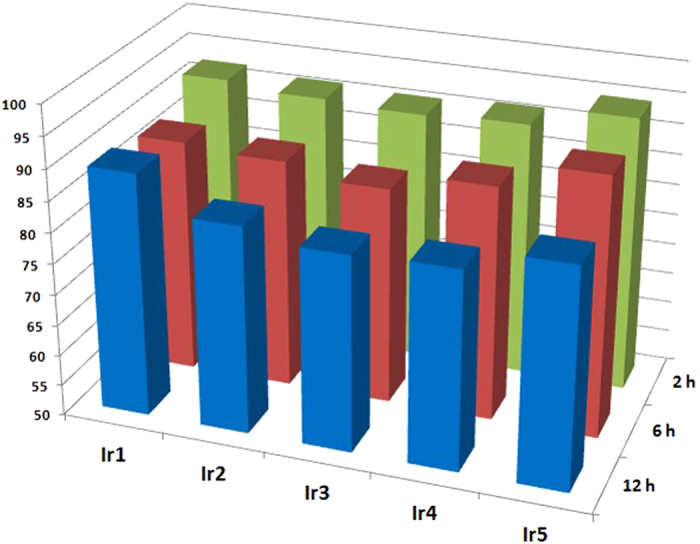
Viability of HeLa cells incubated with 500 nM **Ir1–Ir5**.

An ideal cellular probe for practical applications should minimally perturb living systems at the employed concentrations^27^. In order to evaluate the cytotoxicity of these complexes, HeLa cells were incubated with Ir1-Ir5 and their viability were subsequently tested by MTT assay^43^. The time-dependent effects of Ir1-Ir5 on the cell viability at 37 °C are depicted in Fig. 3. The results show that all complexes exhibit low toxicities. More than 85% of the cells survived after a 12 h incubation with the indicated agents respectively. The very low cytotoxicities of Ir1-Ir5 at work concentration lead us to the conclusion that Ir1-Ir5 are suitable for biological application under the applied conditions (500 nM of complex incubated for 8 min).

### Cellular uptake and localization

To demonstrate that Ir1-Ir5 can specifically accumulate in mitochondria, we performed mitochondrial co-localization imaging experiments with these complexes and MitoTracker Green (MTG), a commercially available mitochondrial co-localizing dye. Unlike [Ru(dip)_2_(dppz)]^2+^ which suffers luminescence quenching in the presence of serum, Ir1-Ir5 showed no obvious luminescent changes when the incubated HeLa cells were exposed to fetal bovine serum (FBS)^44^. This property is consistent with another series of iridium complexes previously reported by our group and convenient for biological applications^36^.

**Figure 4:**
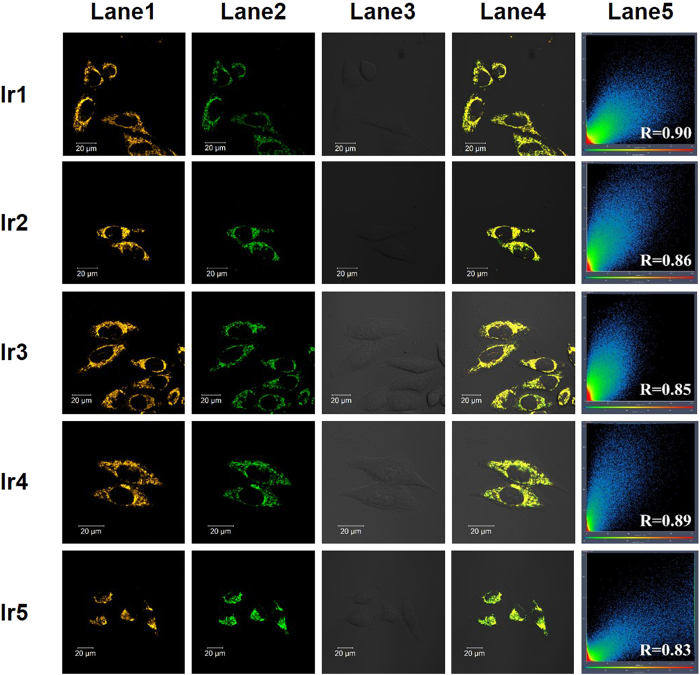
Confocal phosphorescence images and their images overlaid with bright-field images of living HeLa cells incubated with 500 nM of Ir1-Ir5 in DMEM with 10% FBS (pH = 7.4) for 8 min at 37 °C followed by 100 nM of MTR. Lane 1, confocal phosphorescence images of **Ir1-Ir5**; Lane 2, confocal phosphorescence images of MTR; Lane 3, Bright field; Lane 4, overlay of lane 1, lane 2 and lane 3; Lane 4, the overlap coefficient of columns lane 1 and lane 2, and Pearson’s co-localization coefficients are also presented. Excitation wavelength: 405 nm (for all Ir(III) complexes), 488 nm (for MTG); emission filter: 590 ± 30 nm (for all Ir(III) complexes ) and 520 ± 20 nm (for MTG).

HeLa cells were incubated with 500 nM Ir1-Ir5 in Dulbecco minimum essential media (DMEM) containing 10% FBS for 8 min, followed by the removal of the medium, washed with PBS for three times and subsequently incubated with 100 nM MTG for 40 min. As shown in Fig. 4, we can observe an excellent agreement between the orange fluorescence channel from Ir(III) complexes and the green fluorescence channel from MTG, suggesting that Ir1–Ir5 selectively accumulate in mitochondria. The overlap extent of these two channels can be characterized by Pearson’s coefficient^45^. Pearson’s colocalization coefficients were recorded to be 0.90, 0.86, 0.85, 0.89 and 0.83 for Ir1-Ir5, respectively.

**Figure 5:**
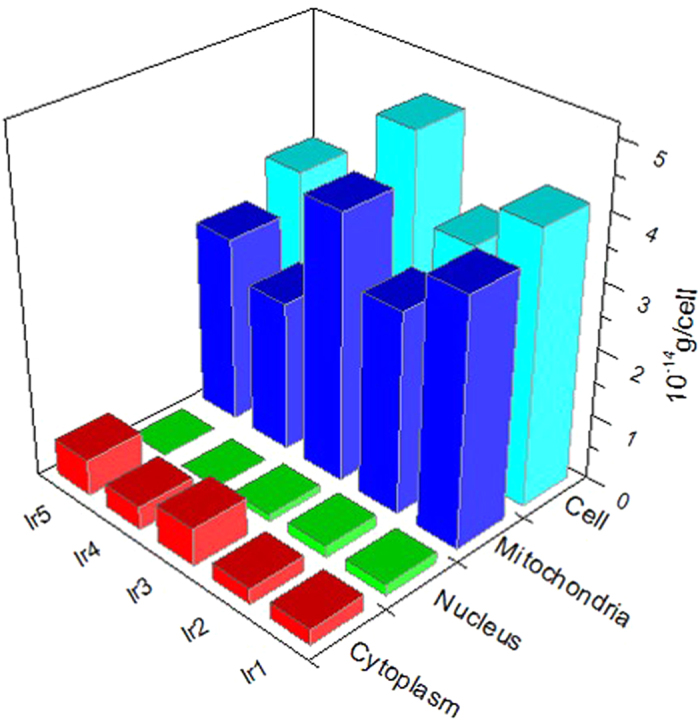
Distribution analysis of Ir1–Ir5 in HeLa cells using ICP-MS. HeLa cells were incubated with **Ir1-Ir5** (500 nM) for 8 min.

Since iridium is not a kind of fundamental element of cells, accurate cellular uptake levels of Ir(III) can be quantitatively determined by using ICP-MS^46^. The ICP-MS experiments were performed for each Ir1-Ir5 complex at a staining concentration of 500 nM to confirm the subcellular distribution of these Ir(III) complexes. The ICP-MS result (Fig. 5) revealed that high levels of cell Ir (nuclei+cytoplasm) were higher than 80%, remarkably, the ratios for Ir1 surpassed 90%. To rationally determine our incubation time, the time-dependent cellular uptake of Ir1 was studied using real-time imaging and ICP-MS, and the results (Figs S21 and S22) showed that the uptake of the Ir(III) complexes reached saturation within 8 min. Considering the systematic error of our experiments and the structural difference among the five complexes, we can infer that incubation time ranging from 5 to 10 min should be suitable for imaging. For convenience of operation in experiment and comparability of these five Ir(III) complexes, we choose 8 min as our incubation time of Ir(III) complexes in all experiments mentioned in this paper. The imaging results revealed that at a low concentration (500 nM), all of these complexes quickly crossed the membrane (within 8 min) and selectively accumulated in the mitochondria.

### Mechanisms of cellular uptake

Small molecules enter cells via energy independent (diffusion, passive diffusion) or energy dependent (endocytosis, active transport) pathways^26^. To explore the mechanism of cellular entry, Ir1 was chosen to investigate cellular uptake at varying temperatures as well as different metabolic and endocytic levels.

**Figure 6:**
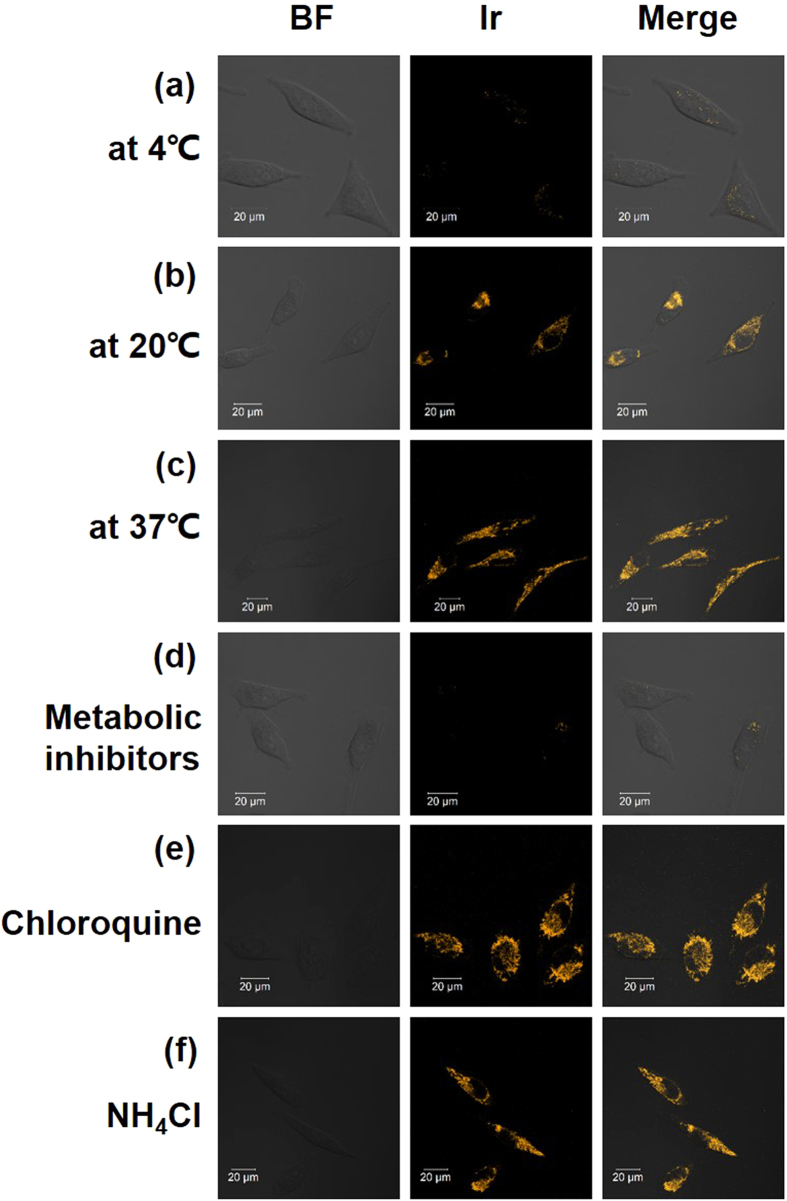
Confocal luminescence image and bright-field images of living HeLa cells incubated with 500 nM Ir1 in DMSO–PBS (pH 7.4, 1: 50, v/v) under different conditions. (**a**–**c**) The cells were incubated with 500 nM **Ir1** at 4 °C, 20 °C and 37 °C for 8 min, respectively. (**d**) The cells were preincubated with 50 mM 2-deoxy-D-glucose and 5 μM oligomycin in PBS for 1 h at 37 °C and then incubated with 500 nM **Ir1** at 37 °C for 8 min. (**e,f**) The cells were pretreated with endocytic inhibitors (chloroquine (50 μM) and NH_4_Cl (50 mM), respectively) and then incubated with 500 nM **Ir1** at 37 °C for 8 min (λ_ex_ = 405 nm, λ_em_ = 590 ± 30 nm).

Blockage of cellular uptake was observed when the cell was incubated at 4 °C or pretreated with metabolic inhibitors (2-deoxy-D-glucose and oligomycin)^44^,^47^. As shown in Fig. 6a,d, the intracellular luminescence was too weak to be visualized. In addition, when the incubation temperature was elevated to room temperature (Fig. 6b), the intracellular luminescence recovered substantially but remained weaker than that at 37 °C (Fig. 6c). From these observations, we deduced that energy appears to play a vital role in the plasma membrane crossing and localizing in the mitochondria of Ir1. The activity of several proteins that aid Ir1 to engage transmembrane transport might be temperature dependent.

Endocytic membrane trafficking is an energy dependent general cell entry mechanism that involves receptor signaling (including signaling from receptor tyrosine kinases and G protein-coupled receptors)^48^. Herein, two kinds of inhibitors of endocytosis (chloroquine and ammonium chloride) were utilized to take a closer look into the procedure of cellular uptake of Ir1^49^. The confocal image revealed that endocytosis inhibition by chloroquine or ammonium chloride does not interfere with membrane transport of Ir1 (Fig. 5e,f). Furthermore, these results were supported by the flow cytometry experiments results (Fig. S16).

Based on previous reports, entry via the endocytotic pathway should be a slow process, thus, these Ir(III) complexes do not require a long time period to enter the cell, which reduces the possibility of endocytotic mechanisms of cellular uptake^50^,^51^. The time-dependent ICP-MS results indicated that Ir1 uptake reached a maximum within 8–10 min (Fig. S21). All of the results suggest that the endocytotic pathway is not responsible for the cellular uptake of Ir1. However, the inherent mechanism about which proteins or other biomolecules assist Ir1 to cross the plasma membrane and accumulate in mitochondria was still under investigation. In summary, Ir1 crosses the plasma membrane using a non-endocytic energy dependent active transport. In addition, based on the structural similarity of the five Ir(III) complexes, we presume other complexes may share the same mechanisms of transmembrane transport.

### Photobleaching and pH-dependent luminescent stability experiments

**Figure 7:**
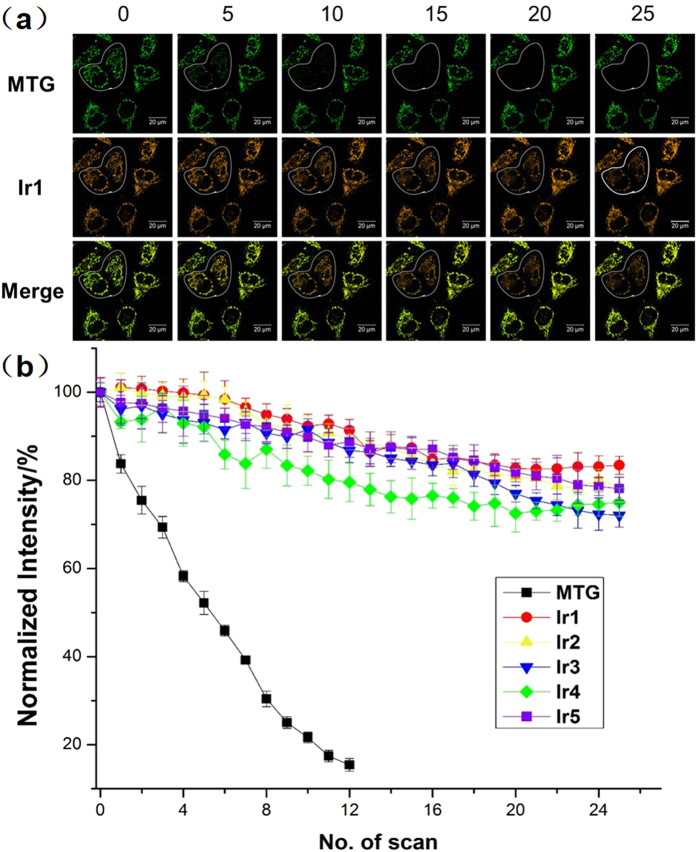
Photobleaching experiments of the Ir(III) complexes in HeLa cells. (**a**) Time-dependent confocal imaging of **Ir1**/MTG co-stained HeLa cells. Confocal images of HeLa cells stained with **Ir1** and MTG before and after verified scans of light irradiation. Time interval per scan: 15 s. Scale bar: 20 mm (λ_ex_ = 405 nm, λ_em_ = 590 ± 30 nm). (**b**) Quantitative photobleaching results indicate that **Ir1–Ir5** exhibited robust emission intensity under continuous light irradiation.

Photostability (anti-bleaching property) is a crucial criteria to evaluate new fluorescent imaging agents for bioimaging, especially for real-time and long-term imaging^12^. The comparison in photostabilities of all five Ir(III) complexes and the organic dye (MTG) under continuous laser irradiation was made by the photobleaching experiment of Ir(III) complexes and MTG co-stained HeLa cells. Using Ir1 versus MTG as an example, during 10 scans with a total irradiation time of ~150 s, luminescence in the bleaching area was significantly decreased in the MTG channel, while no obvious change in the Ir1 channel could be observed (Fig. 7a). Even after 25 scans with a total irradiation time of 6 min, approximately 20% of the fluorescent signal was lost for Ir1 but the mitochondria in the HeLa cells were still clearly observed. Similar results of Ir2-Ir5 were shown in Figs S24–S27. Statistically, after 10 scans, an ~80% decrease in the normalized fluorescence signal intensity of MTG was detected and in the contrary, less than 10% intensity decrease were observed in the Ir(III) complexes (Fig. 7b). This good photobleaching stability may be due to an inherent property of heavy-metal complexes as well as the formation of AIE particles because the condensed particles can prevent further photobleaching and photooxidation by avoiding oxygen diffusion into the particles^12^,^52^.

For bioimaging, dyes should be usable within the physiological pH range^41^. We therefore investigated the luminescence properties of the Ir(III) complexes in the pH range of 4.0–10.0. The results indicated that the luminescence of Ir2-Ir5 barely changed when the pH value was in the range of 4.0–8.0. However, as shown in Fig. S28, when the pH value is higher than 9.0, the photoluminescence intensity decreased substantially (~30%). These results are different from those previously reported even though the Ir(III) complexes have similar ligands^36^. Therefore, the difference may be due to the introduction of the triphenylamine group. It is important to note that the stability in a pH range from 4.0 to 8.0 is also favorable for mitochondria because their physiological pH is 6.50 to 8.20^53^.

### Real-time monitoring of the mitophagy process

**Figure 8:**
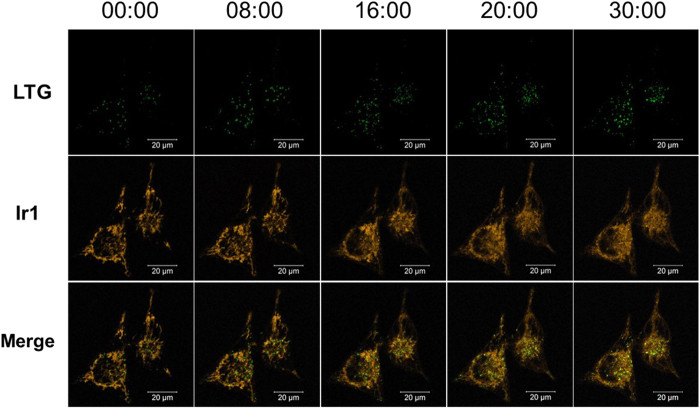
Phosphorescence images of CCCP (10 μM) treated living HeLa cells stained with Ir1 (0.5 μM). To inhibit autophagic flux, the cells were preincubated with chloroquine (50 μM) prior to the addition of CCCP. Scale bar: 20 mm (λ_ex_ = 405 nm, λ_em_ = 590 ± 30 nm).

**Figure 9:**
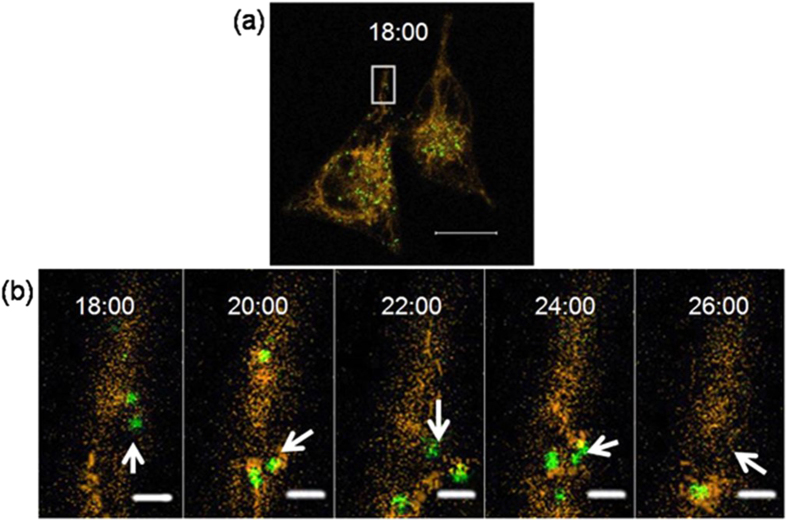
Confocal images of HeLa cells stained with Ir1 (500 nM, orange) and LTG (100 nM, green) in the presence of CCCP (10 μM). (**a**) Time points (min) were selected from the onset and completion of the mitophagy process. The regions (**b**) indicated in white boxes are enlarged from the shown area of this cell. Following imaging is shown in (**b**) until the observed mitophagy process is completed in the selected area. Scale bar: 20 μm (**a**) and 2 μm (**b**). (**Ir1**, λ_ex_ = 405 nm, λ_em_ = 590 ± 30 nm).

Mitochondria continuously oxidize substrates and maintain a proton gradient across the lipid bilayer with a very large membrane potential of ~180 mV^54^,^55^. Variation in the membrane potential of mitochondria in cells has evolved as a read-out of the mitochondrial functional status and generates signals to activate pathways that repair or eliminate defective mitochondria^2^. Twig *et al.* demonstrated that mitochondria are prone to mitophagy when the pool of mitochondria suffer a significant reduction in the mitochondrial membrane potential^56^. As an ionophore, CCCP was used as an important agent for gaining insight into the mitophagy pathway^57^,^58^. In addition, CCCP cannot only be used to trigger mitophagy but also bulk autophagy^59^,^60^. Therefore, in this study, the HeLa cells were treated with the CCCP to trigger mitophagy, and Ir1 and LysoTracker Green (LTG) were used to locate the mitochondria and lysosome, respectively. In addition, chloroquine was added to inhibit autophagic flux according to the previously reported methods^8^. Along with reticulum-like mitochondria being gradually transformed into small and dispersed fragments upon exposure to CCCP, the intensity of green photoluminescence signal from LTG increased sharply, which indicate the occurrence of mitophagy (Fig. 8 and Video S1). At 20 min of real-time monitoring, a new green fluorescent spot appears (white arrow) and overlaps the orange Ir1-labelled mitochondria. This result indicates the formation of acidic autophagosome and initiation of mitophagy process in this area^17^. The disappearance of the green fluorescent signal at 26 min suggests that the mitophagy process was complete in this area of the cell (Fig. 9). The time range of completing mitophagy observed in the present study is similar to previous reports^17^,^61^. Additionally, a long-term observation experiment was performed without the addition of CCCP to determine if the mitophagy process was induced by CCCP (Fig. S29). In this observation, the fluorescence signals from the mitochondria and lysosomes were mostly unchanged except for a decrease in the signal due to photobleaching. The result indicates that the observed mitophagy is primarily due to the presence of CCCP.

## Conclusion

In summary, five cyclometalated iridium(III) complexes (**Ir1**–**Ir5**) exhibiting AIE properties were designed and synthesized. As AIE phosphorescent agents, these Ir(III) complexes were employed as mitochondrial probes with good photostability. In a short imaging time period (8 min) at a low concentration (500 nM) with no phosphorescent intensity fluctuation in the mitochondrial physiological range, **Ir1-Ir5** selectively and efficiently located the mitochondria. Similar to Ir(III) complexes in our previous study, the results from the cellular uptake experiments indicated that these Ir(III) complexes cross the cellular membrane by a non-endocytotic active transport approach. Of high interest, **Ir1**, along with LTG, were employed to successfully monitor mitophagy induced by CCCP. With this convenient and competent mitophagy probe, we can tackle the problem occurred in mitophagy tracking including not only the short-time dynamics change but also pH fluctuation, violent morphology alteration and membrane potential lost. This mitochondrion-specific probe expands the molecule libraries of AIE cyclometalated iridium(III) complexes probes and is expected to be a useful tool for a range of biological imaging, dynamic monitoring studies and helping to give an insight into the mitophagy process involved in disease.

## Methods

### General Procedure

All of the reactants and solvents were purchased from commercial sources and used as received unless otherwise stated. All of the procedures involving IrCl_3_·xH_2_O were carried out under an argon atmosphere in dark.

All Microanalysis (C, H, and N) was carried out using an Elementar Vario EL elemental analyzer. ^1^H NMR spectra were recorded using a 300 MHz nuclear magnetic resonance spectrometer (Varian, Mercury-Plus 300) and a 400 MHz Bruker nuclear magnetic resonance spectrometer (AVANCE III). All of the chemical shifts are reported relative to tetramethylsilane (TMS). The electronic absorption spectra were recorded using a Perkin-Elmer Lambda 850 UV/Vis spectrometer. The emission spectra were recorded using a Perkin-Elmer LS 55 luminescence spectrometer. The electrospray mass spectra were recorded using Shimadzu liquid chromatography-mass spectrometry (LCMS-2010A). The inductively coupled plasma mass spectrometry (ICP-MS) experiments were performed on a Thermo X2 instrument. Confocal luminescent microscopy experiments were conducted on a LSM 710 (Carl Zeiss) laser scanning confocal microscope. The particle size analyses were determined at room temperature on a zeta potential analyzer (MALVERN HPPS5001, England).

### Synthesis of ligands and its corresponding iridium(III) complexes

1,10-phenanthroline-5,6-dione^62^ as well as [Ir(ppy)_2_Cl]_2_^63^ was synthesized according to published methods.

### Synthesis of N,N-diphenyl-4-(1-phenyl-1H-imidazo[4,5-*f*][1,10]phenanthrolin-2-yl)aniline (dpipa)

A mixture of 1,10-phenanthroline-5,6-dione (0.525 g, 2.5 mmol), ammonium acetate (3.85 g, 50 mmol), 4-(N,N-diphenylamino)benzaldehyde (0.683 g, 2.5 mmol), aniline (0.233 g, 2.5 mmol) and glacial acetic acid (15 ml) was refluxed under argon for 15 h. Then, the reaction mixture was cooled to room temperature and poured into water (40 ml). The obtained mixture was neutralized with 25% NH_3_ aqueous solution and extracted with chloroform (20 ml × 4). The organic phase was combined and dried with MgSO_4_ overnight, and then solvent was removed under vacuum. The crude product was purified by column chromatography on silica with CH_2_Cl_2_-ethanol (50:1, v/v) as the eluent. The resulting solid was recrystallized from chloroform-toluene to afford a pale yellow microcrystal. Yield, 0.951 g, 70.6%. Anal. Calcd. for C_37_H_25_N_5_ (%): C, 82.35; H, 4.67; N, 12.98. Found (%): C, 82.14; H, 4.85; N, 12.91. ^1^H NMR (300 MHz, DMSO-*d6*): δ 9.11–8.87 (m, 3H), 7.91–7.81 (m, 1H), 7.74 (s, 5H), 7.44 (s, 3H), 7.32 (d, *J* = 7.8 Hz, 5H), 7.04 (d, *J* = 7.8 Hz, 6H), 6.80 (s, 2H). ES-MS (CH_2_Cl_2_): m/z = 540.5 [M+H]^+^.

### Synthesis of N,N-diphenyl-4-(1-(p-tolyl)-1H-imidazo[4,5-*f*][1,10]phenanthrolin-2-yl)aniline (dtipa)

This compound was synthesized using a procedure that was identical to that described for ligand **dpipa** except that p-toluidine (0.268 g, 2.5 mmol) was used instead of aniline. Yield, 0.908 g, 65.7%. Anal. Calcd. for C_26_H_18_N4 (%): C, 82.29; H, 5.09; N, 12.63. Found (%): C, 82.43; H, 5.20; N, 12.37. ^1^H NMR (300 MHz, CDCl_3_): δ 9.25 (d, *J* = 5.1 Hz, 2H), 9.08 (d, *J* = 4.2 Hz, 1H), 7.85–7.75 (m, 1H), 7.52–7.40 (m, 7H), 7.30–7.23 (m, 6H), 7.14–7.05 (m, 5H), 6.95 (d, *J* = 8.7 Hz, 2H), 2.58 (s, 3H). ES-MS (CH_2_Cl_2_): m/z = 554.4 [M+H]^+^.

### Synthesis of N,N-diphenyl-4-(tert-butyl)phenyl-1H-imidazo[4,5-*f*][1,10]phenanthrolin-2-yl)aniline (dbipa)

This compound was synthesized using a procedure that was identical to that described for ligand **dpipa** except that 4-(tert-butyl)aniline (0.373 g, 2.5 mmol) was used instead of aniline. Yield, 1.093 g, 73.5%. Anal. Calcd. for C_41_H_33_N_5_ (%): C, 82.66; H, 5.58; N, 11.76. Found (%): C, 82.39; H, 5.73; N, 11.88. ^1^H NMR (300 MHz, DMSO): δ 9.09–9.02 (m, 1H), 9.00–8.93 (m, 1H), 8.94–8.88 (m, 1H), 7.89–7.78 (m, 1H), 7.67 (d, *J* = 13.8 Hz, 4H), 7.46 (d, *J* = 9.0 Hz, 3H), 7.32 (t, *J* = 7.5 Hz, 5H), 7.10 (s, 2H), 7.03 (d, *J* = 7.5 Hz, 4H), 6.82 (d, *J* = 9.0 Hz, 2H), 1.41 (s, 9H). ES-MS (CH_2_Cl_2_): m/z = 596.5 [M+H]^+^.

### Synthesis of N,N-diphenyl-4-fluorophenyl-1H-imidazo[4,5-*f*][1,10]phenanthrolin-2-yl)aniline (dfipa)

This compound was synthesized using a procedure that was identical to that described for ligand **dpipa** except that 4-fluoroaniline (0.278 g, 2.5 mmol) was used instead of aniline. Yield, 1.050 g, 72.8%. Anal. Calcd for C_37_H_24_FN_5_ (%): C, 79.70; H, 4.34; N, 12.56. Found (%): C, 79.46; H, 4.67; N, 12.68. ^1^H NMR (300 MHz, DMSO): δ 9.10–8.87 (m, 3H), 7.89–7.80 (m, 3H), 7.60–7.42 (m, 5H), 7.34 (t, *J* = 7.8 Hz, 5H), 7.15–7.01 (m, 6H), 6.85 (d, *J* = 8.7 Hz, 2H). ES-MS (CH_2_Cl_2_): m/z = 558.4 [M+H]^+^.

### Synthesis of N,N-diphenyl-4-phenoxyphenyl-1H-imidazo[4,5-*f*][1,10]phenanthrolin-2-yl)aniline (dpoipa)

This compound was synthesized using a procedure that was identical to that described for ligand **dpipa** except that 4-phenoxyaniline (0.462 g, 2.5 mmol) was used instead of aniline. Yield, 0.959 g, 60.7%. Anal. Calcd for C_43_H_29_N_5_O (%): C, 81.75; H, 4.63; N, 11.08. Found (%): C, 81.51; H, 4.82; N, 11.27. ^1^H NMR (300 MHz, CDCl_3_): δ 9.06 (s, 1H), 8.96 (t, *J* = 7.2 Hz, 2H), 7.85 (dd, *J* = 8.1, 4.2 Hz, 1H), 7.74 (d, *J* = 8.4 Hz, 2H), 7.55 (d, *J* = 4.2 Hz, 1H), 7.47 (dd, *J* = 16.8, 8.1 Hz, 5H), 7.37–7.26 (m, 6H), 7.20 (t, *J* = 8.1 Hz, 3H), 7.13 (d, *J* = 7.2 Hz, 2H), 7.07 (d, *J* = 8.1 Hz, 4H), 6.88 (d, *J* = 8.4 Hz, 2H). ES-MS (CH_2_Cl_2_): m/z = 632.5 [M+H]^+^.

The five Ir(III) complexes were synthesized as described below. A chloro-bridged dimer [Ir(ppy)_2_Cl]_2_ (0.088 g, 0.08 mmol) and corresponding ligands (0.015 mmol) were placed in a 100 ml round-bottom flask with 40 ml of methanol and CHCl_3_ (1:1, v/v). The mixture was heated at 60 °C for 8 h under argon and avoids to be exposed to light. After the solution cooled to room temperature, the solvent was removed under vacuum to afford an orange precipitate. The product was purified by column chromatography on alumina using acetonitrile-ethanol (10:1, v/v) as the eluent.

### Ir1

Yield: 0.1127 g, 72.2%. Anal. Calcd. for C_59_H_41_N_7_ClIr (%): C, 65.87; H, 3.84; N, 9.12. Found (%): C, 65.67; H, 4.09; N, 8.91. ^1^H NMR (400 MHz, DMSO): δ9.30 (dd, *J* = 8.4, 1.4 Hz, 1H), 8.25 (m, 3H), 8.15 (dd, *J* = 8.4, 5.2 Hz, 1H), 8.06 (dd, *J* = 5.2, 1.2 Hz, 1H), 7.99–7.86 (m, 4H), 7.84–7.72 (m, 6H), 7.50 (dd, *J* = 7.2, 5.2 Hz, 4H), 7.46–7.41 (m, 1H), 7.36 (t, *J* = 8.0 Hz, 4H), 7.15 (t, *J* = 7.2 Hz, 2H), 7.10–7.05 (m, 5H), 7.05–6.91 (m, 5H), 6.85 (d, *J* = 8.8 Hz, 2H), 6.28 (dd, *J* = 13.4, 7.2 Hz, 2H). ES-MS (CH_3_OH): m/z = 1040.25 [M-Cl^−^]^+^.

### Ir2

Yield: 0.1222 g, 74.7%. Anal. Calcd for C_60_H_43_N_7_ClIr (%): C, 66.13; H, 3.98; N, 9.00. Found (%): C, 66.01; H, 4.17; N, 8.82. ^1^H NMR (400 MHz, DMSO): δ9.29 (d, *J* = 8.2 Hz, 1H), 8.31–8.21 (m, 3H), 8.14 (dd, *J* = 8.2, 5.2 Hz, 1H), 8.06 (d, *J* = 4.4 Hz, 1H), 7.93 (m, 4H), 7.77 (dd, *J* = 8.8, 5.2 Hz, 1H), 7.68 (d, *J* = 8.8 Hz, 2H), 7.59–7.48 (m, 7H), 7.37 (t, *J* = 8.0 Hz, 4H), 7.15 (t, *J* = 7.2 Hz, 2H), 7.11–6.99 (m, 8H), 6.95 (dd, *J* = 16.0, 8.0 Hz, 2H), 6.85 (d, *J* = 8.8 Hz, 2H), 6.28 (dd, *J* = 11.2, 7.6 Hz, 2H). ES-MS (CH_3_OH): m/z = 1054.00 [M-Cl^−^]^+^.

### Ir3

Yield: 0.1123 g, 66.2%. Anal. Calcd. for C_63_H_49_N_7_ClIr (%): C, 66.86; H, 4.36; N, 8.66. Found (%): C, 66.58; H, 4.65; N, 8.53. ^1^H NMR (400 MHz, DMSO): δ 9.29 (dd, *J* = 8.4, 1.6 Hz, 1H), 8.28 (t, *J* = 9.2 Hz, 2H), 8.23 (dd, *J* = 5.2, 1.6 Hz, 1H), 8.14 (dd, *J* = 8.4, 5.2 Hz, 1H), 8.07 (dd, *J* = 5.2, 1.2 Hz, 1H), 7.96 (dd, *J* = 10.4, 8.4 Hz, 2H), 7.93–7.86 (m, 2H), 7.74 (m, 5H), 7.52–7.46 (m, 5H), 7.36 (dd, *J* = 10.8, 5.2 Hz, 4H), 7.14 (t, *J* = 7.6 Hz, 2H), 7.10–7.00 (m, 8H), 6.99–6.91 (m, 2H), 6.88–6.81 (m, 2H), 6.28 (dd, *J* = 9.6, 7.6 Hz, 2H), 1.39 (s, 9H). ES-MS (CH_3_OH): m/z = 1096.25 [M-Cl^−^]^+^.

### Ir4

Yield: 0.116 g, 81.7%. Anal. Calcd. for C_59_H_40_N_7_ClIr (%): C, 65.94; H, 3.75; N, 9.12. Found (%): C, 65.76; H, 3.88; N, 8.98. ^1^H NMR (400 MHz, DMSO): δ 9.29 (d, *J* = 8.4 Hz, 1H), 8.31–8.25 (m, 2H), 8.23 (d, *J* = 5.2 Hz, 1H), 8.17–8.13 (m, 1H), 8.08 (d, *J* = 4.8 Hz, 1H), 7.99–7.93 (m, 2H), 7.89 (dd, *J* = 13.6, 7.6 Hz, 4H), 7.81 (dd, *J* = 7.6, 5.6 Hz, 1H), 7.64–7.54 (m, 3H), 7.50 (d, *J* = 7.2 Hz, 4H), 7.37 (t, *J* = 7.2 Hz, 4H), 7.15 (t, *J* = 7.2 Hz, 2H), 7.12–6.99 (m, 8H), 6.96 (dd, *J* = 15.6, 7.6 Hz, 2H), 6.88 (d, *J* = 7.2 Hz, 2H), 6.28 (dd, *J* = 13.2, 7.6 Hz, 2H). ES-MS (CH_3_OH): m/z = 1058.20 [M-Cl^−^]^+^.

### Ir5

Yield: 0.13 g, 77.5%. Anal. Calcd for C_65_H_45_N_7_OClIr (%): C, 66.85; H, 3.88; N, 8.40. Found (%): C, 66.68; H, 4.14; N, 8.21. ^1^H NMR (400 MHz, DMSO): δ 9.29 (dd, *J* = 8.4, 1.2 Hz, 1H), 8.28 (t, *J* = 9.2 Hz, 2H), 8.23 (dd, *J* = 5.2, 1.6 Hz, 1H), 8.14 (dd, *J* = 8.4, 5.2 Hz, 1H), 8.10 (dd, *J* = 5.2, 1.2 Hz, 1H), 7.97 (t, *J* = 8.4 Hz, 2H), 7.93–7.84 (m, 3H), 7.82–7.76 (m, 2H), 7.66 (dd, *J* = 8.4, 1.2 Hz, 1H), 7.55 (d, *J* = 8.8 Hz, 2H), 7.51–7.43 (m, 4H), 7.40–7.29 (m, 6H), 7.26 (d, *J* = 7.2 Hz, 1H), 7.23–7.14 (m, 4H), 7.12–7.08 (m, 4H), 7.08–6.90 (m, 8H), 6.29 (dd, *J* = 10.0, 8.0 Hz, 2H). ES-MS (CH_3_OH): m/z = 1131.95 [M-Cl^−^]^+^.

### Cell viability assay

The HeLa cells were cultivated in DMEM with 10% FBS (Gibco) and 1% antibiotic solution (1% penicillin and streptomycin) (Gibco) at 37 °C under 5% CO_2_ and 95% relative humidity. The cytotoxicity of these Ir(III) complexes against HeLa cells was evaluated using the MTT assay. The exponentially grown HeLa cells were seeded in triplicate into 96-well plates at an appropriate concentration. After incubation for 24 h, the cells were treated with **Ir1-Ir5** respectively at the concentration used for staining (500 nM) for various durations. To stain the live cells, 10 μl of MTT (5 mg/ml, Sigma) was added to each well. Then, the cells were incubated for an additional 4 h at previous culture conditions. The media was carefully removed, and then, 150 μl of DMSO was added to every sample well to dissolve the formed formazan crystals. The optical density of the obtained formazan solution of the samples was measured at 590 nm using an ELISA reader (BioTek Instruments Inc., Winooski, Vermont).

### Confocal luminescence imaging

The HeLa cells were incubated at a density of ~1 × 10^4^ cells per ml in DMEM supplemented with 10% FBS as well as 1% antibiotic material at 37 °C in a 5% CO_2_. After incubation for 1 day to make sure adhesion of a majority of the cells were accomplished, then cells were directly treated with 500 nM Ir(III) complexes in DMEM for 8 min without any replacement of medium. After removing the DMEM and washing with PBS buffer three times to remove the remaining dye, the HeLa cells were further stained with MTG for another 40 min. The cells were carefully washed three times with PBS buffer prior to the luminescence imaging measurements. Laser power for Ir(III) complexes in the wavelength of 405 nm is 150 μW, and that for MTG in the wavelength of 488 nm is 100 μW The cell images were captured with Zeiss LSM 710 NLO confocal microscope (63 × /NA 1.4 oil immersion objective) and analyzed using the AxioVision 4.2 software (Carl Zeiss).

### Cellular uptake analysis

The HeLa cells were incubated at a density of ~2 × 10^5^ cells per ml in 25 cm^2^ culture plates (Corning) with 6 ml of DMEM with 10% FBS. After incubating for 48 h, the monolayer Hela cells nearly oversperead the whole plates. **Ir1-Ir5** (500 nM) was added to the culture medium and incubated for 8 min or for a time period within the range of 0–30 min at 37 °C. After digestion with trypsin (Gibco) and gathering in 3 ml DMEM, the HeLa cells were counted and divided into two equal parts as tested samples. The first sample for nuclear isolation using a nucleus extraction kit and the second one for mitochondrial extraction using a cytoplasm and mitochondria extraction kit (Shanghai Sangon Biological Engineering Technology & Services Co. Ltd.). All of the treated samples were digested in 65% nitric acid at room temperature for at least 48 h. Each sample was diluted with Mili-Q Water to obtain 3% HNO_3_ sample solutions. The iridium concentration in the two parts for each sample was determined by inductively coupled plasma mass spectrometry.

### Flow cytometry analysis

HeLa cells, at a density of about 1 × 10^5^ cells per ml, were seeded in 6-well plates in DMEM with 10% FBS for 24 h in an incubator and then treated with **Ir1** (500 nM) for 8 min at 37 °C without medium replacement. The cells were washed with PBS three times to remove the residual complexes, trypsinized for 1 min to gain cell suspension. After centrifugation for the cell suspension the supernatant was discarded, then 1 ml PBS was added. These cell uptake samples were analyzed using a FACS Canto II instrument (BD Biosciences, USA).

### Mitophagy real-time monitoring

The HeLa cells were incubated at a proper density until the cells were adhered. According to the literature method^64^, **Ir1** (500 nM) and LTG (100 nM) were used to co-stain HeLa cells, and time-dependent confocal images were collected after mitophagy induction. Mitochondrial uncoupler CCCP (10 μM) was used to induce mitophagy, and chloroquine (50 μM) was used to inhibit autophagic flux. Excitation wavelengths of 405 nm and 488 nm with the varied power (150 μW and 100 μW, respectively). The cell images were captured with Zeiss LSM 710 NLO confocal microscope (63 × /NA 1.4 oil immersion objective) and analyzed using the AxioVision 4.2 software (Carl Zeiss).

## Additional Information

**How to cite this article**: Jin, C. *et al.* Cyclometalated Iridium(III) Complexes as AIE Phosphorescent Probes for Real-Time Monitoring of Mitophagy in Living Cells. *Sci. Rep.* **6**, 22039; doi: 10.1038/srep22039 (2016).

## Supplementary Material

Supplementary Information

Supplementary Video
