# Supplementary material for: Cyclometalated Iridium(III) Complexes as AIE Phosphorescent Probes for Real-Time Monitoring of Mitophagy in Living Cells
Source: Sci Rep. 2016 Feb 24;6:22039. doi: 10.1038/srep22039 (PMC4764980; doi:10.1038/srep22039)
Supplement: Supplementary Information [file srep22039-s1.doc]

**Supporting Information**

**Cyclometalated Iridium(III) Complexes as AIE Phosphorescent Probes For Real-Time Monitoring of Mitophagy in Living Cells**

Chengzhi Jin^1^, Jiangping Liu^1^, Yu Chen^1^, Ruilin Guan^1^, Cheng Ouyang^1^,

Yanjiao Zhu^2^, Liangnian Ji^1^, and Hui Chao^1^*

*^1^ MOE Key Laboratory of Bioinorganic and Synthetic Chemistry, School of Chemistry and Chemical Engineering, Sun Yat-Sen University, Guangzhou 510275, P. R. China*

*E-mail:* [*ceschh@mail.sysu.edu.cn*](mailto:ceschh@mail.sysu.edu.cn)

*^2^ School of Materials Science and Engineering, Hubei University, Wuhan 430062, P. R. China*

Figure S1 ES-MS spectra of Ir1 in CH_3_OH solutions S3

Figure S2 ^1^H NMR spectra of Ir1 in DMSO-*d_6_* S3

Figure S3 ES-MS spectra of Ir2 in CH_3_OH solutions S4

Figure S4 ^1^H NMR spectra of Ir2 in DMSO-*d_6_* S4

Figure S5 ES-MS spectra of Ir3 in CH_3_OH solutions S5

Figure S6 ^1^H NMR spectra of Ir3 in DMSO-*d_6_* S5

Figure S7 ES-MS spectra of Ir4 in CH_3_OH solutions S6

Figure S8 ^1^H NMR spectra of Ir4 in DMSO-*d_6_* S6

Figure S9 ES-MS spectra of Ir5 in CH_3_OH solutions S7

Figure S10 ^1^H NMR spectra of Ir5 in DMSO-*d_6_* S7

Figure S11 UV-Vis spectra of Ir1-Ir5 in DMSO/PBS (v/v = 1:9) solutions S8

Figure S12 Emission spectra of Ir2 in DMSO–PBS mixtures with different water

fractions S9

Figure S13 Emission spectra of Ir3 in DMSO/PBS mixtures with different water

fractions S10

Figure S14 Emission spectra of Ir4 in DMSO/PBS mixtures with different water

fractions S11

Figure S15 Emission spectra of Ir5 in DMSO/PBS mixtures with different water

fractions S12

Figure S16 Size distribution of Ir1 aggregates in DMSO/PBS mixtures with 90% water fraction. S13

Figure S17 Size distribution of Ir2 aggregates in DMSO/PBS mixtures with 90% water fraction. S13

Figure S18 Size distribution of Ir3 aggregates in DMSO/PBS mixtures with 90% water fraction. S14

Figure S19 Size distribution of Ir4 aggregates in DMSO/PBS mixtures with 90% water fraction. S14

Figure S20 Size distribution of Ir5 aggregates in DMSO/PBS mixtures with 90% water fraction. S15

Figure S21 Real-time monitoring of staining with Ir1 by ICP-MS. S16

Figure S22 Real-time monitoring of staining with Ir1 by confocal microscopy. S16

Figure S23 Flow cytometric histogram profile of cellular uptake of Ir1 in HeLa cells. S17

Figure S24 Photobleaching experiments of Ir2 in HeLa cell S18

Figure S25 Photobleaching experiments of Ir3 in HeLa cell S18

Figure S26 Photobleaching experiments of Ir4 in HeLa cell S19

Figure S27 Photobleaching experiments of Ir5 in HeLa cell S19

Figure S28 Emission intensity of 10 μM Ir1-Ir5 at 590 nm under different pH in a Britton-Robinson buffer. S20

Figure S29 Confocal images of HeLa cells stained with Ir1 and LTG without CCCP... S20

Table S1 Photophysical data of Ir1-Ir5 S21

**Movie S1** Real-Time Monitoring of Mitophagy in HeLa cells**.....................................................S21**


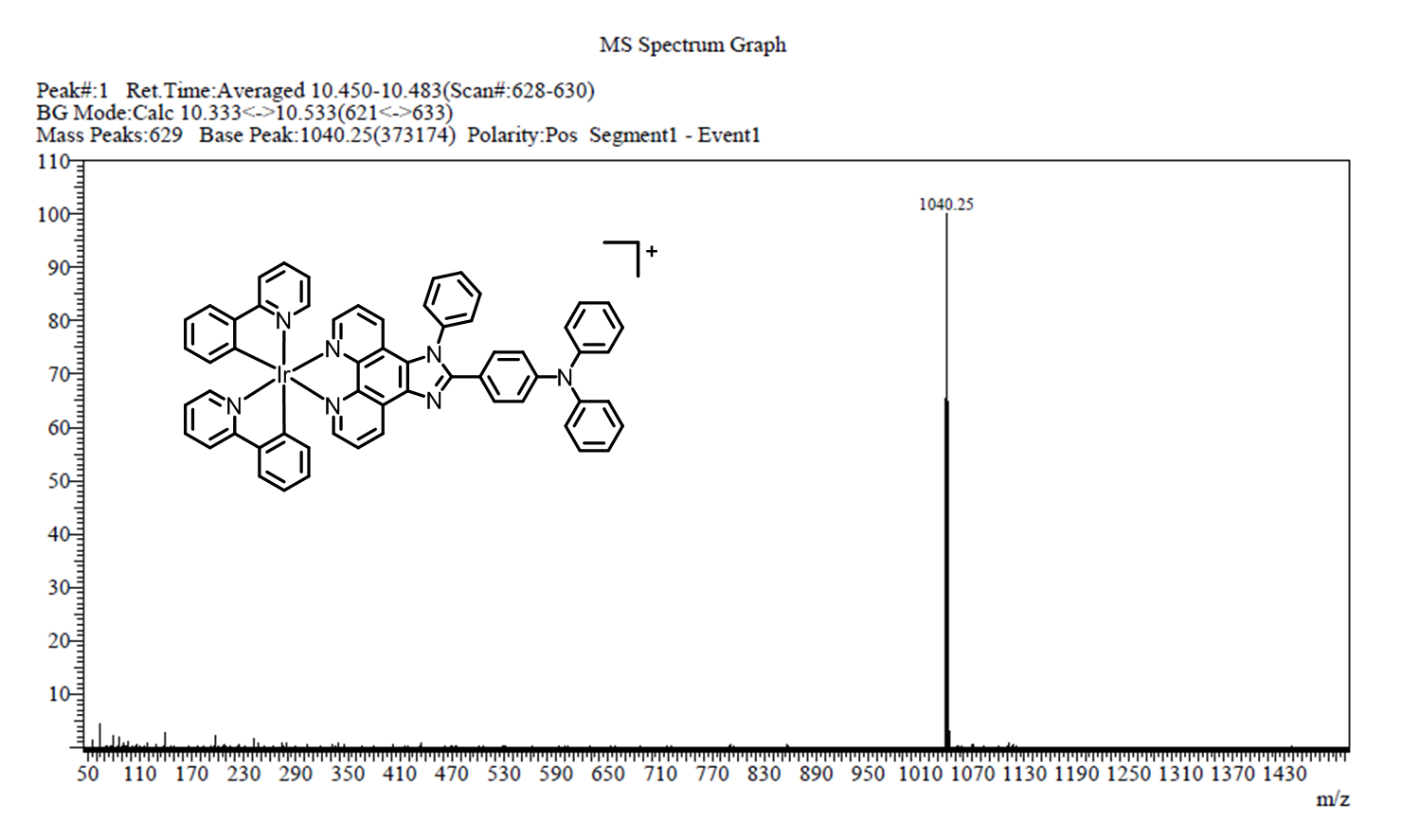


**Figure S1** ES-MS spectra of **Ir1** in CH_3_OH solutions.


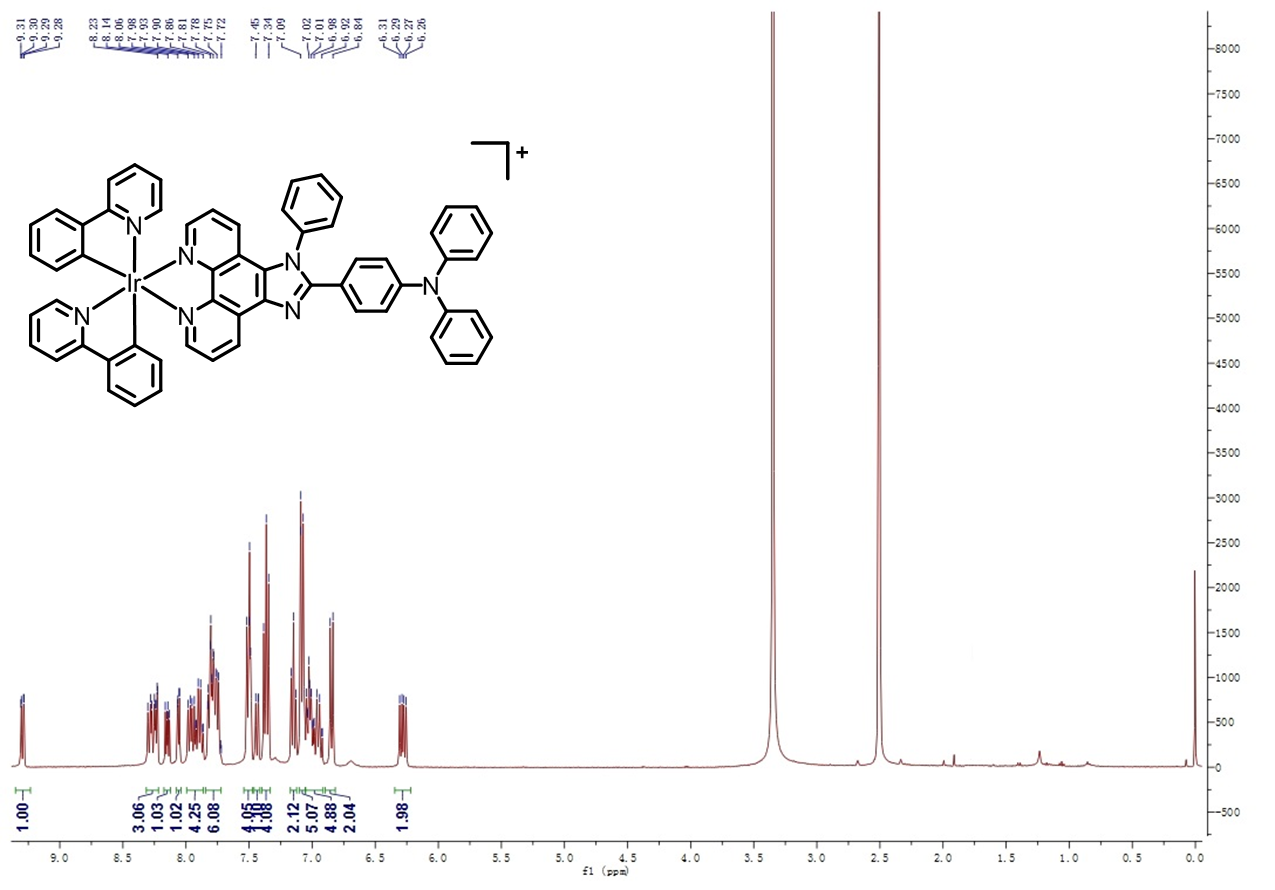


**Figure S2** ^1^H NMR (400 MHz) spectra of **Ir1** in DMSO-*d_6_*.


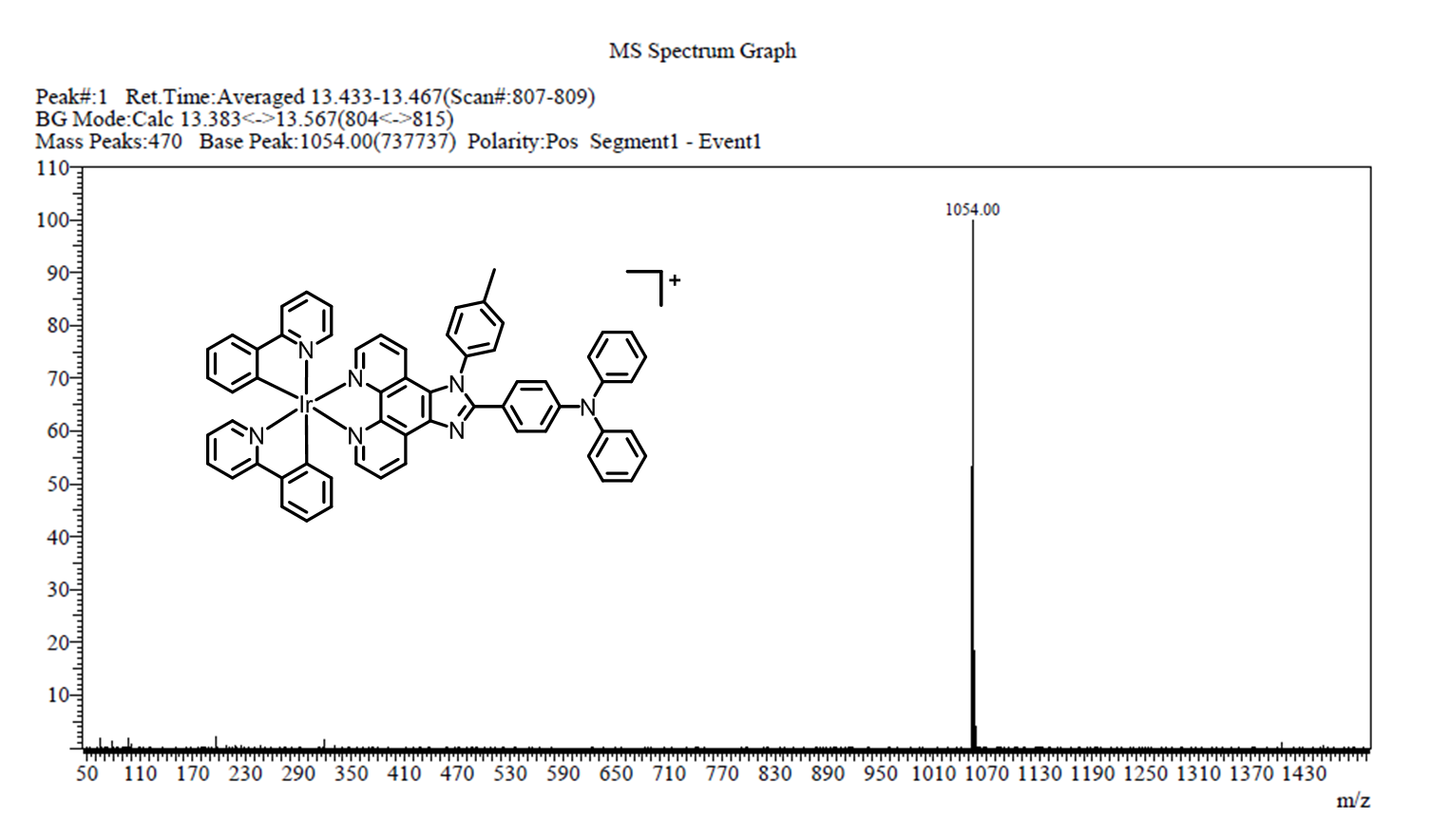


**Figure S3** ES-MS spectra of **Ir2** in CH_3_OH solutions.


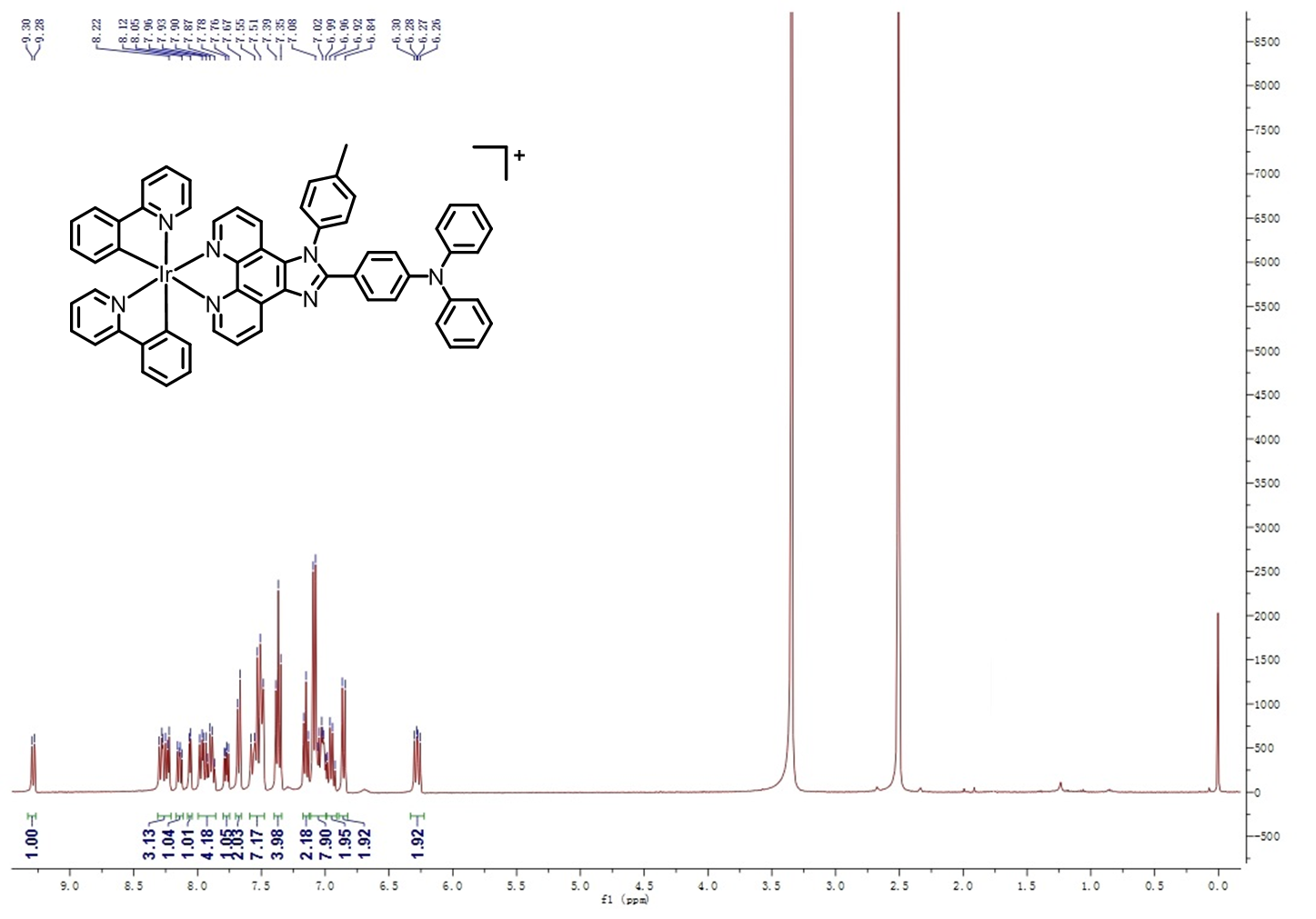


**Figure S4** ^1^H NMR (400 MHz) a spectra of **Ir2** in DMSO-*d_6_*.


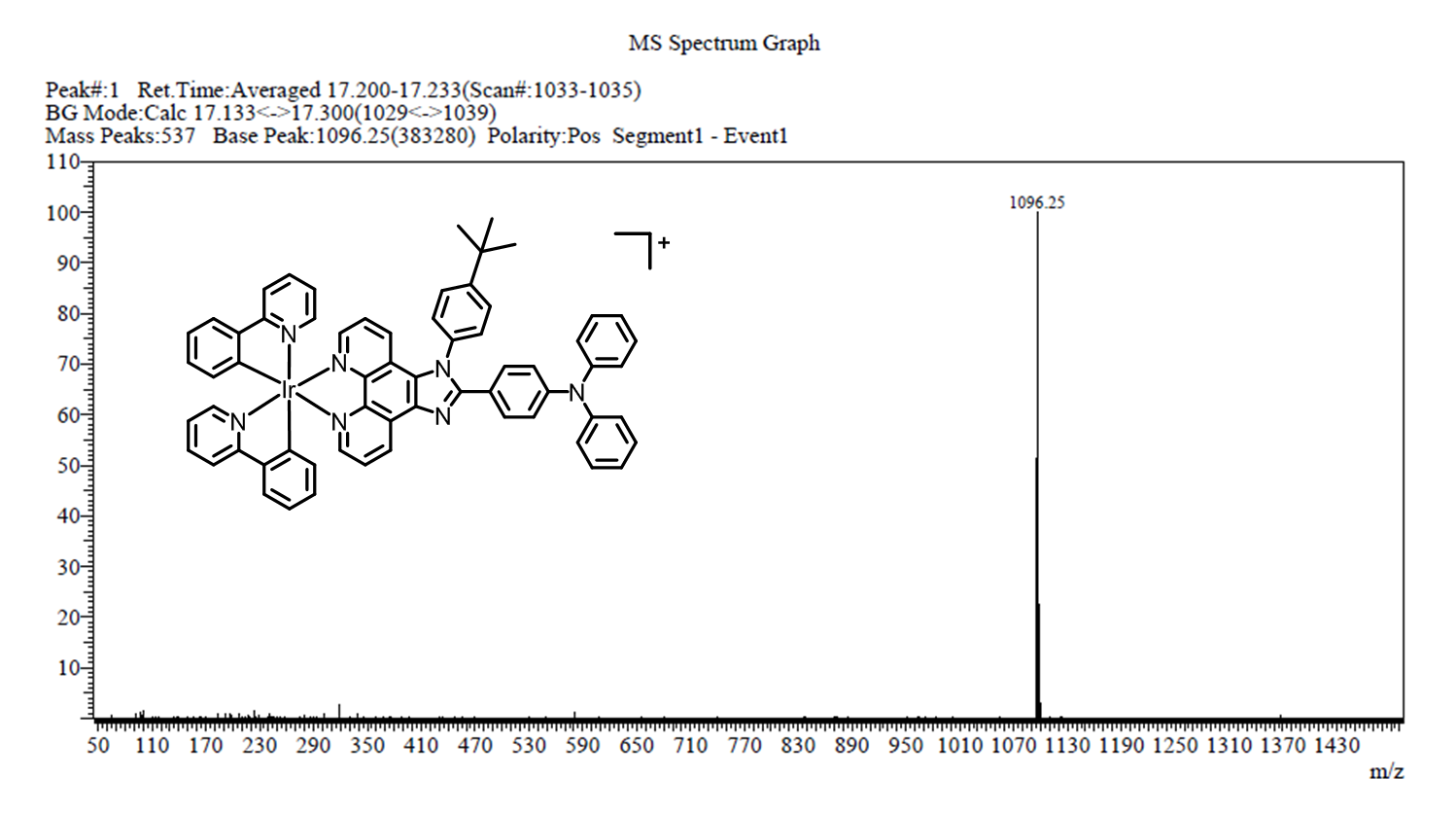


**Figure S5** ES-MS spectra of **Ir3** in CH_3_OH solutions.

**
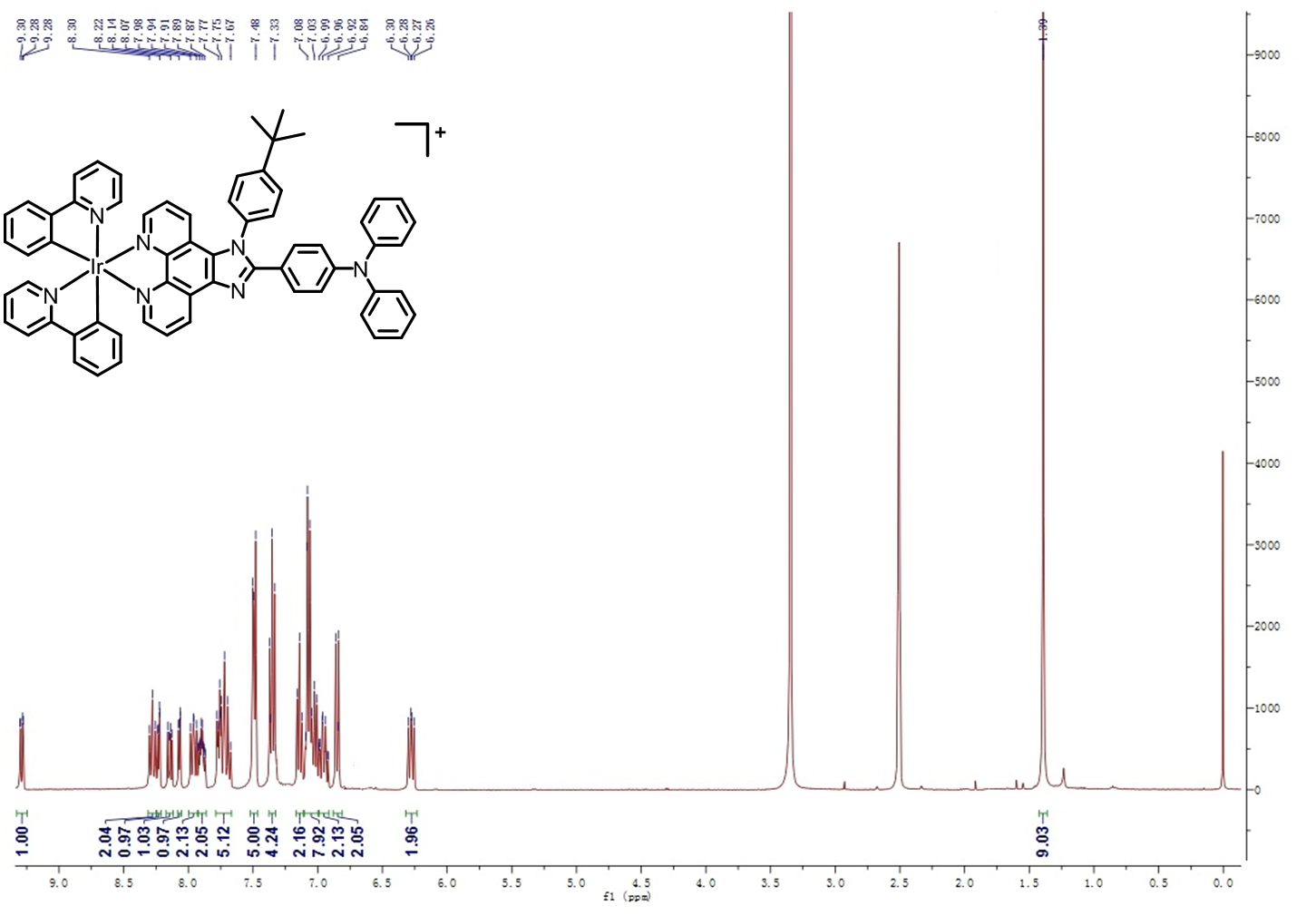
**

**Figure S6** ^1^H NMR (400 MHz) spectra of **Ir3** in DMSO-*d_6_*.


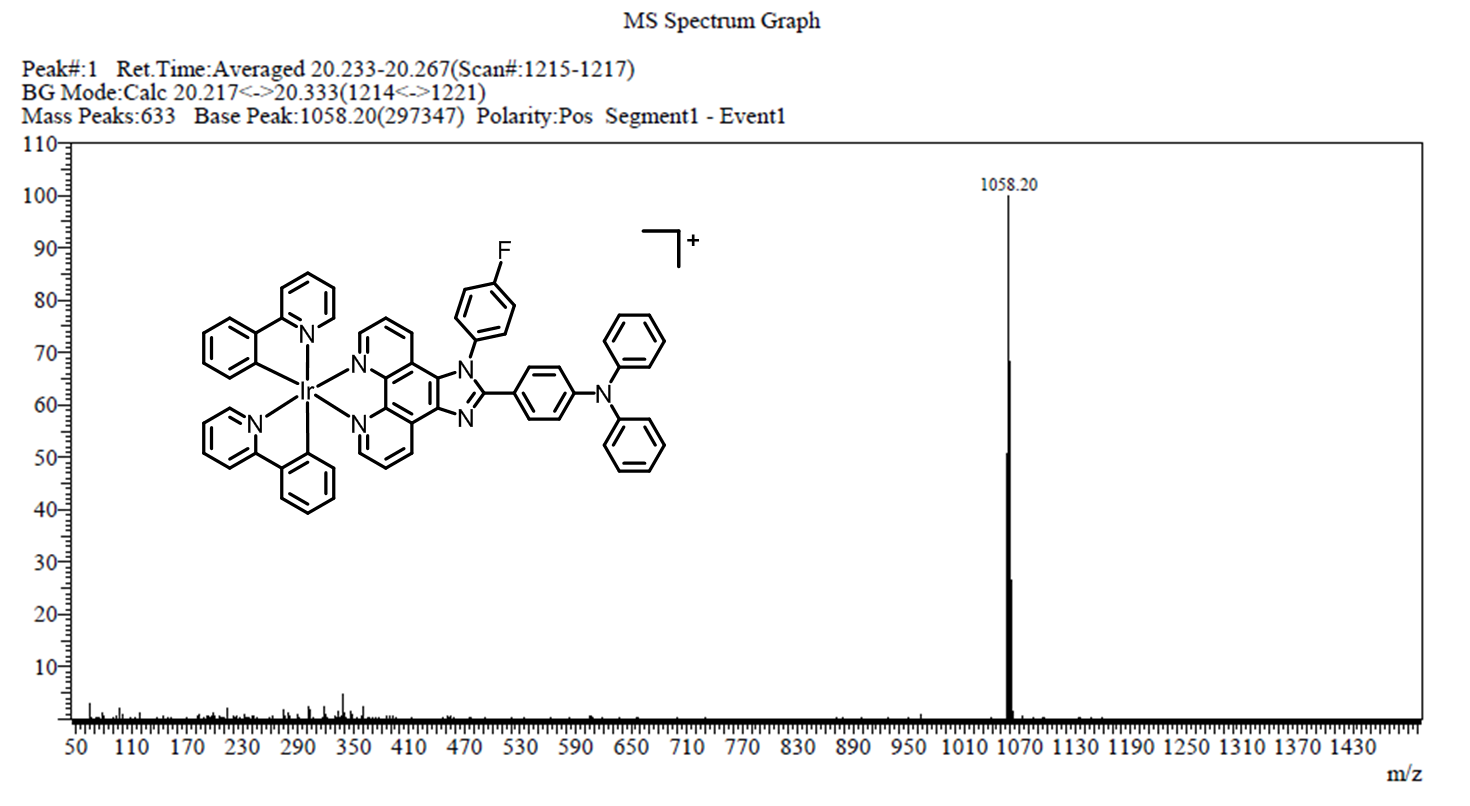


**Figure S7** ES-MS spectra of **Ir4** in CH_3_OH solutions.


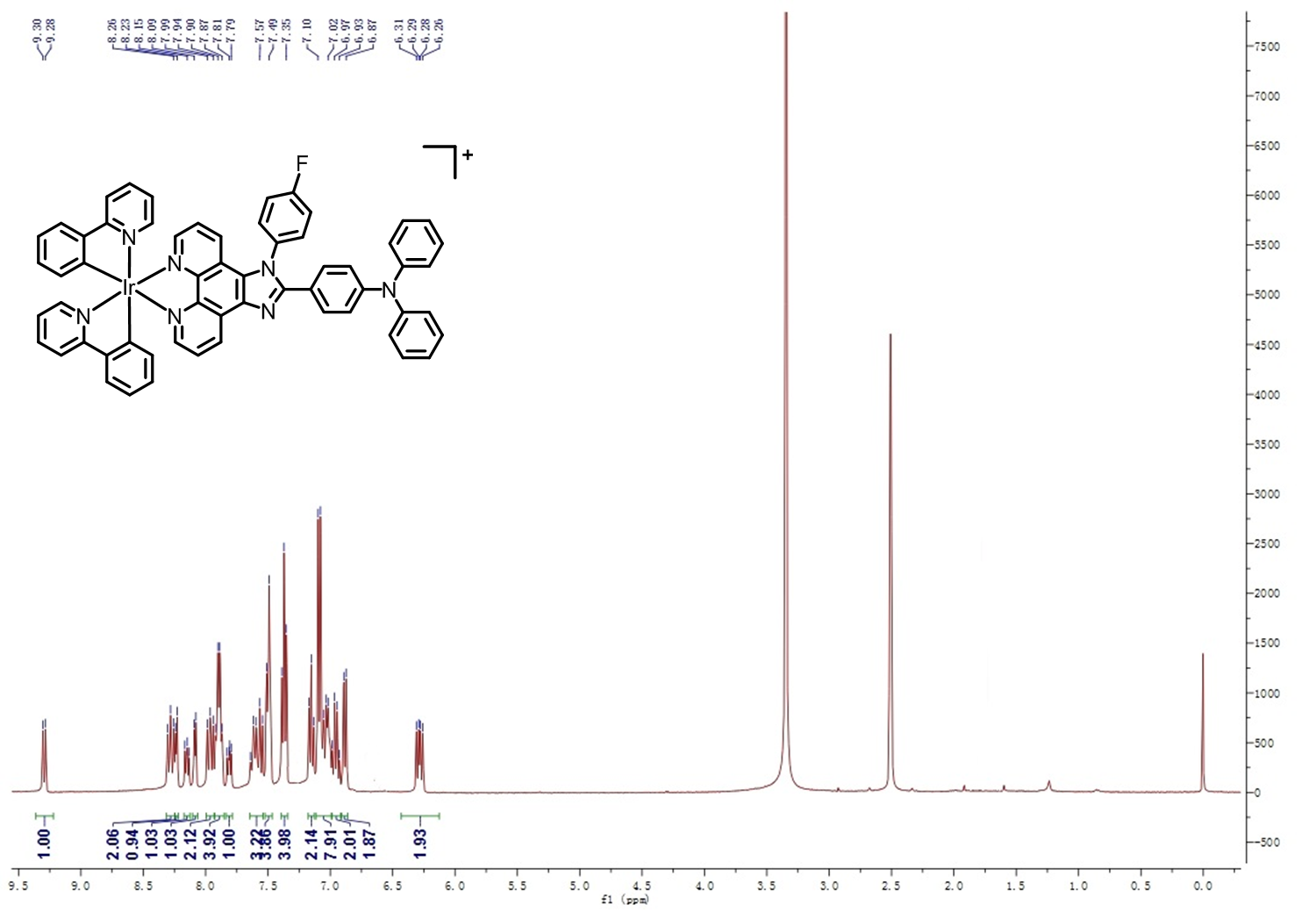


**Figure S8** ^1^H NMR (400 MHz) spectra of **Ir4** in DMSO-*d_6_*.


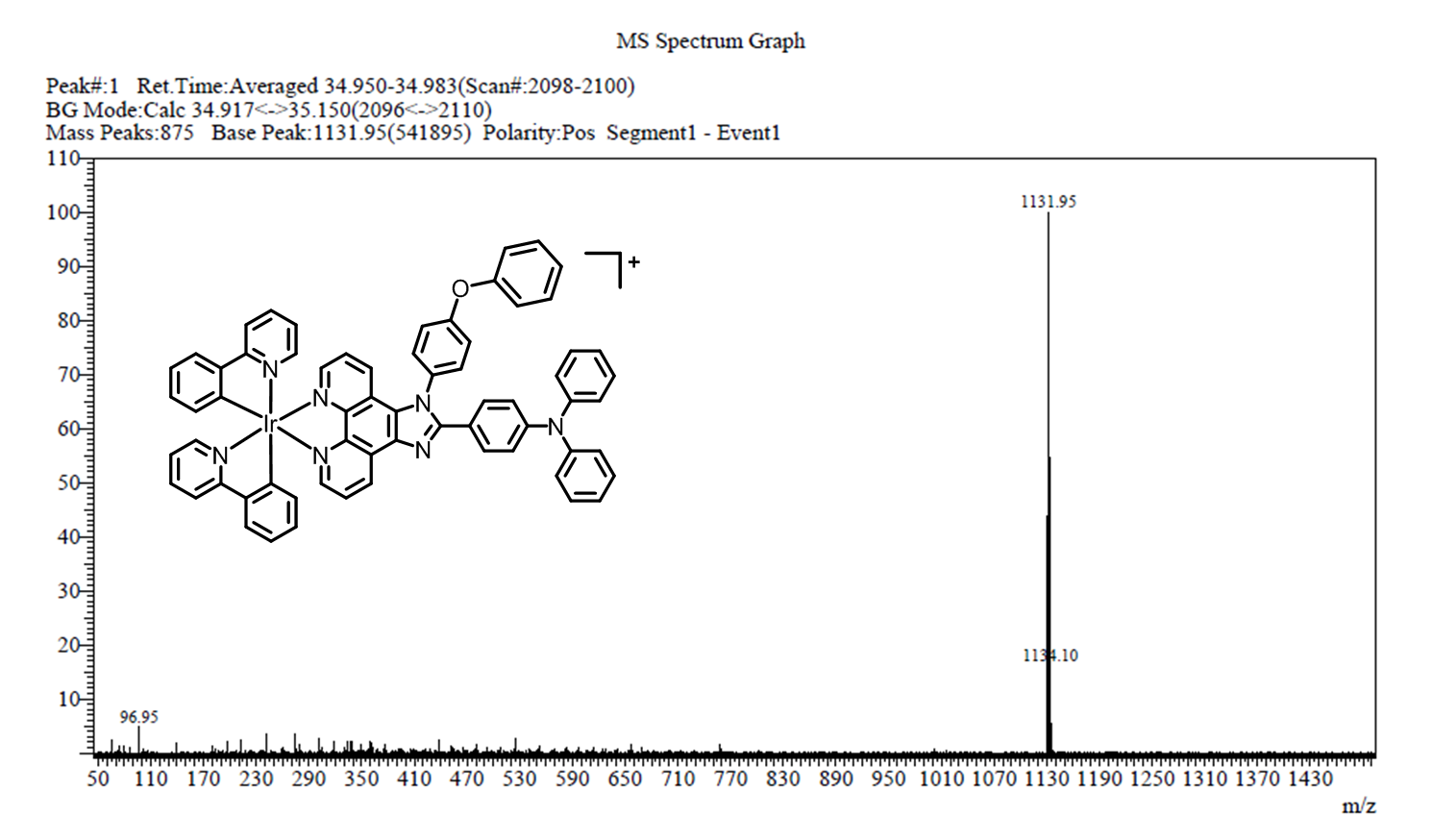


**Figure S9** ES-MS spectra of **Ir5** in CH_3_OH solutions.

**
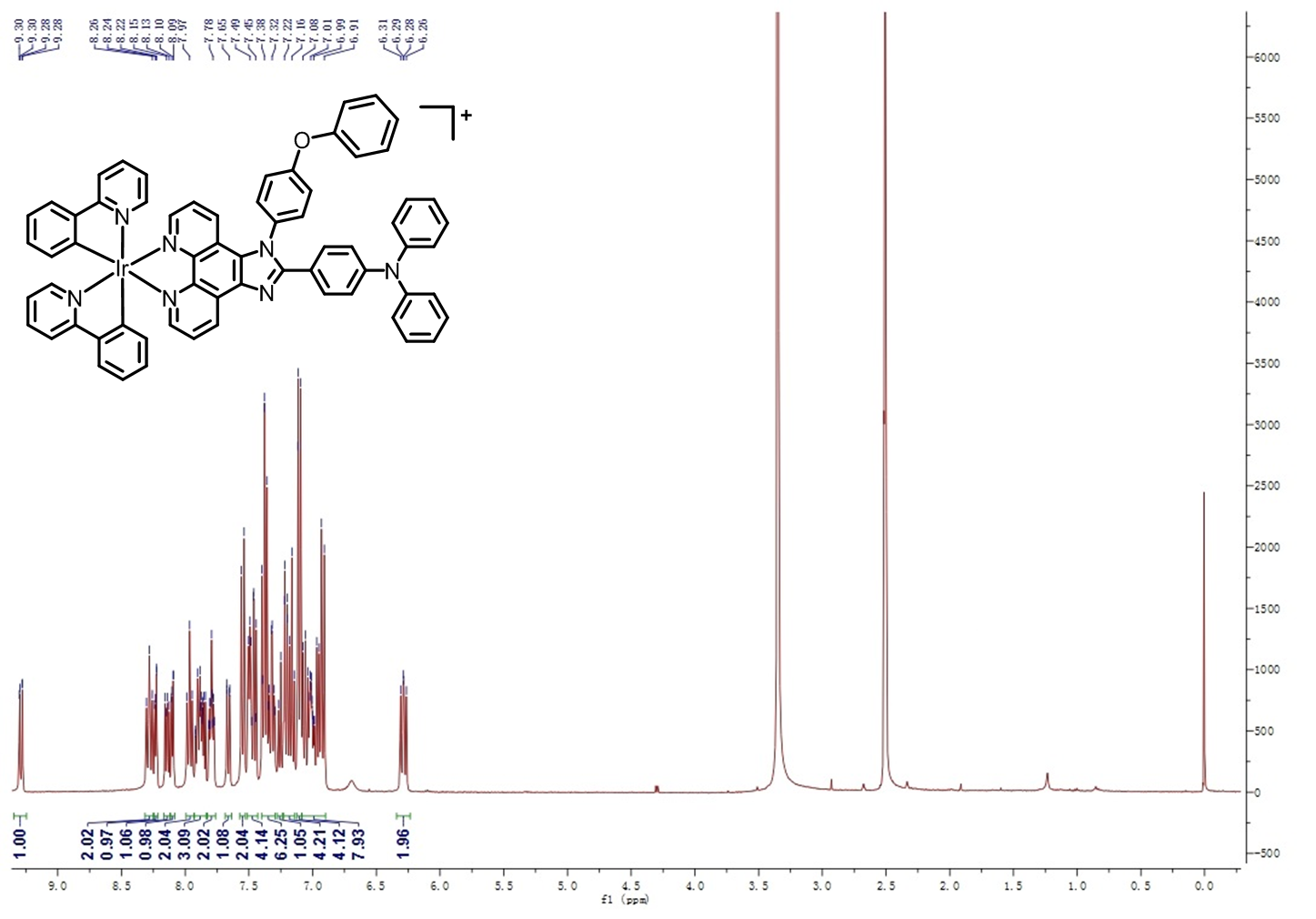
**

**Figure S10** ^1^H NMR (400 MHz) spectra of **Ir5** in DMSO-*d_6_*.


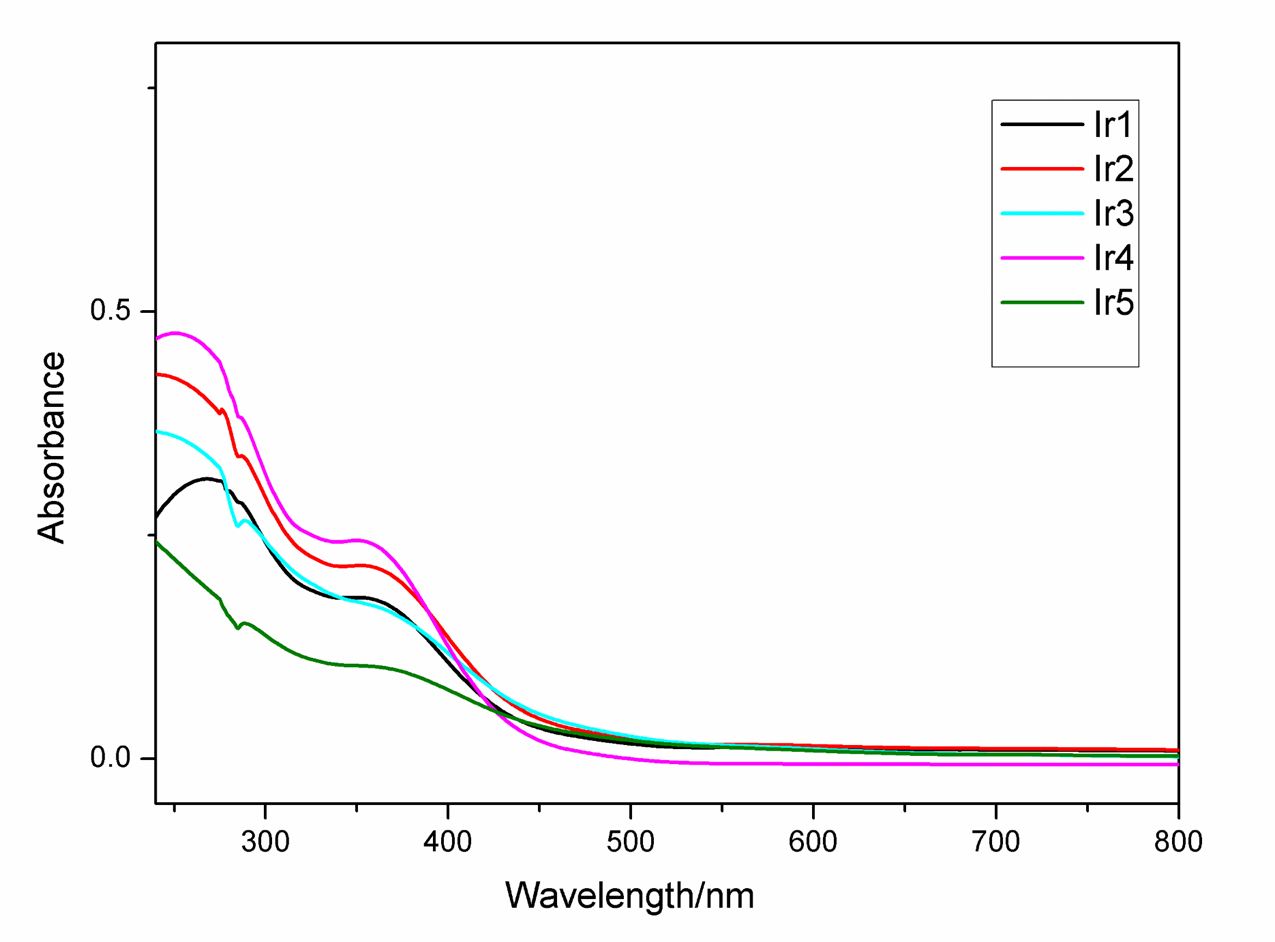


**Figure S11** UV-Vis spectra of **Ir1-Ir5** (10 μM) in DMSO/PBS (v/v = 1:9) solutions at 298K.


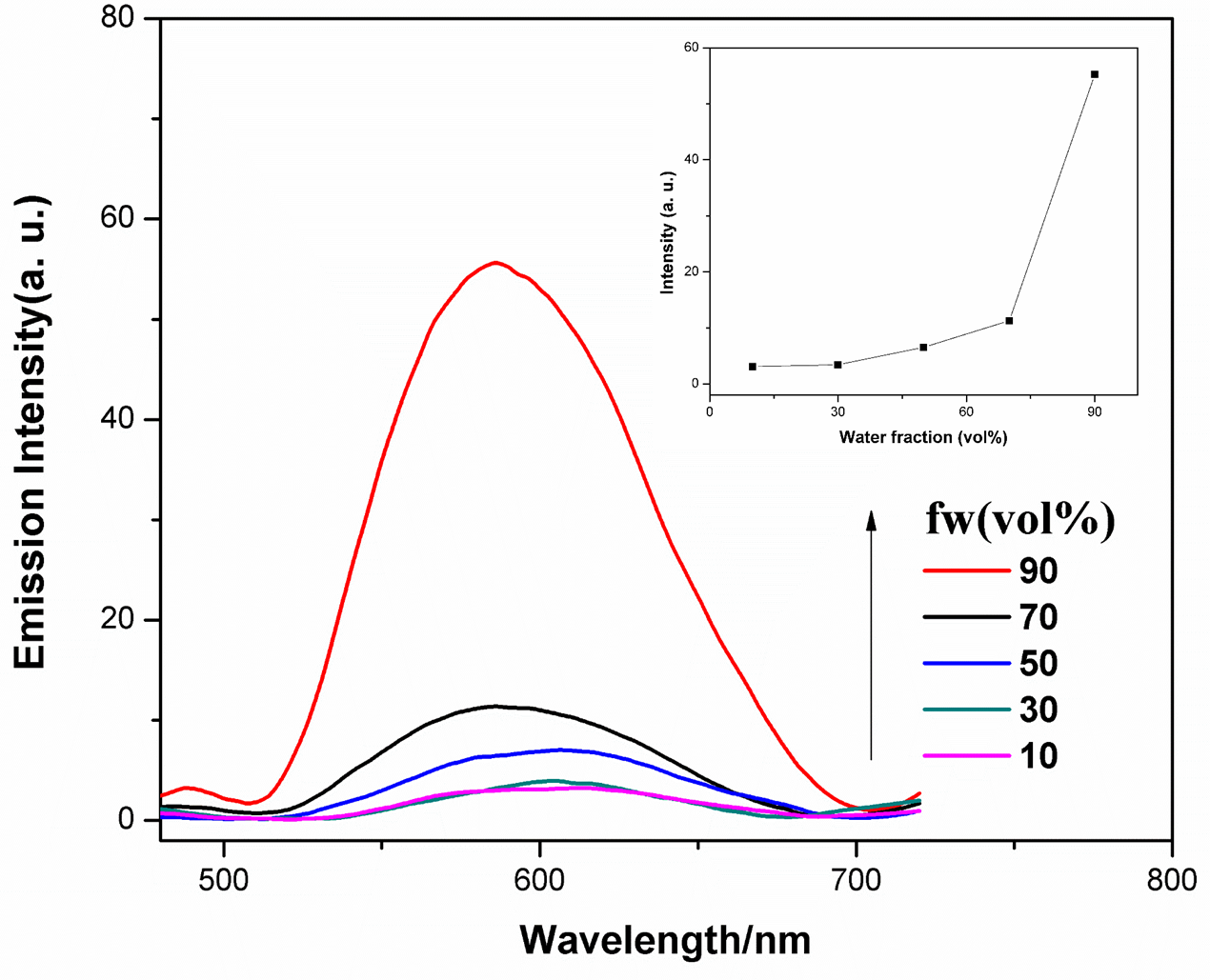


**Figure S12** Emission spectra of **Ir2** in DMSO/PBS mixtures with different water

fractions at 298K with an excitation wavelength of 381 nm.


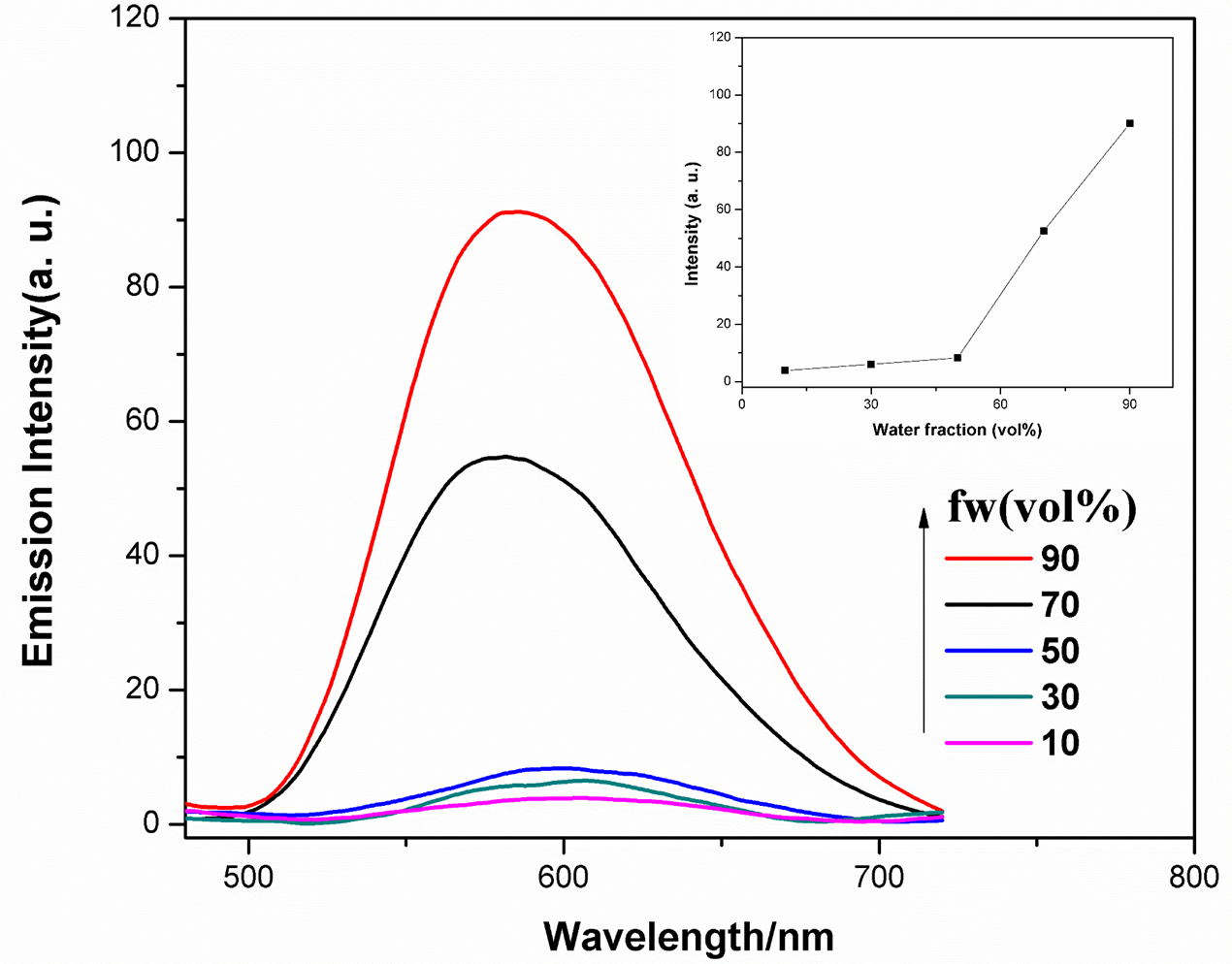


**Figure S13** Emission spectra of **Ir3** in DMSO/PBS mixtures with different water

fractions at 298K with an excitation wavelength of 381 nm.


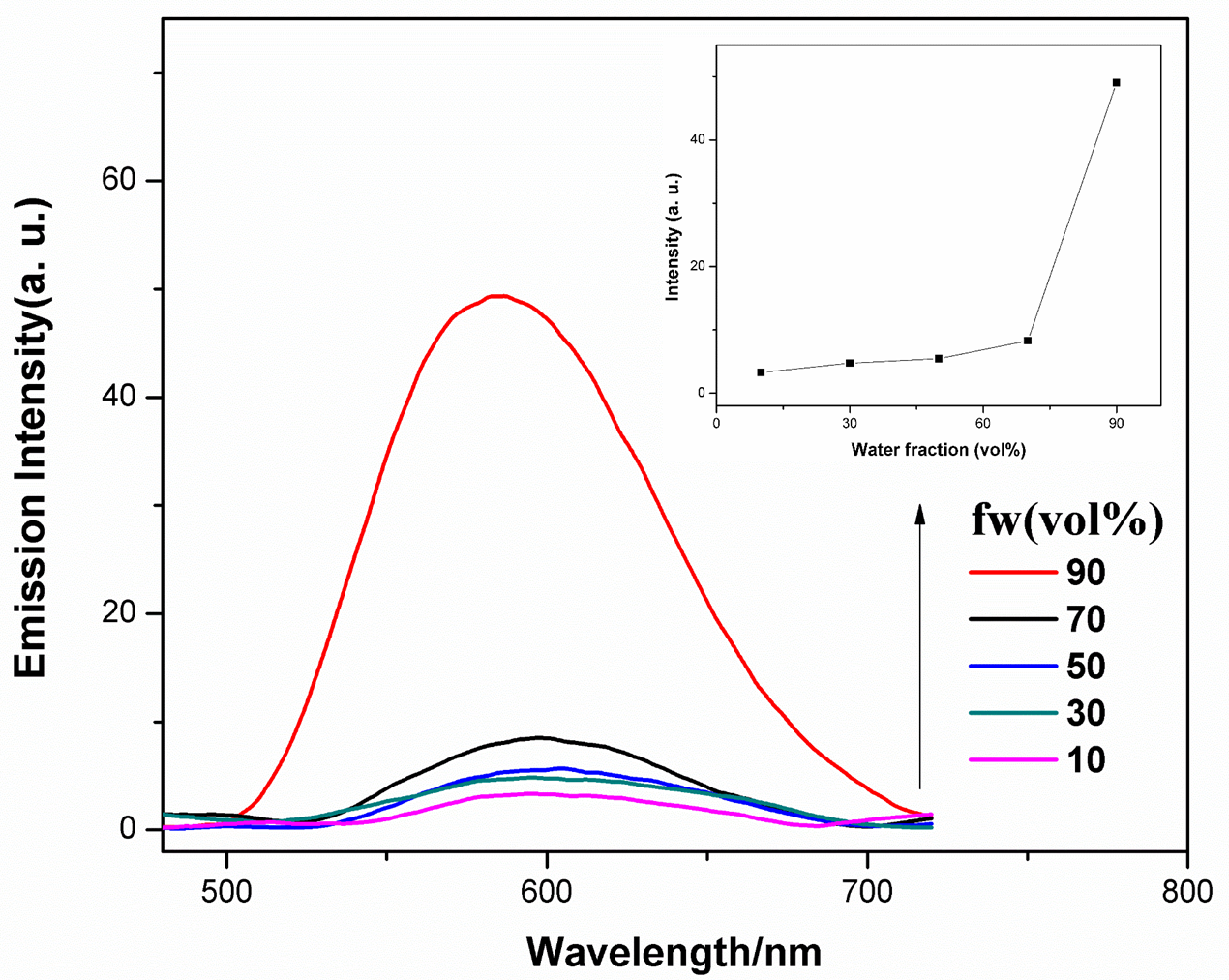


**Figure S14** Emission spectra of **Ir4** in DMSO/PBS mixtures with different water

fractions at 298K with an excitation wavelength of 381 nm.


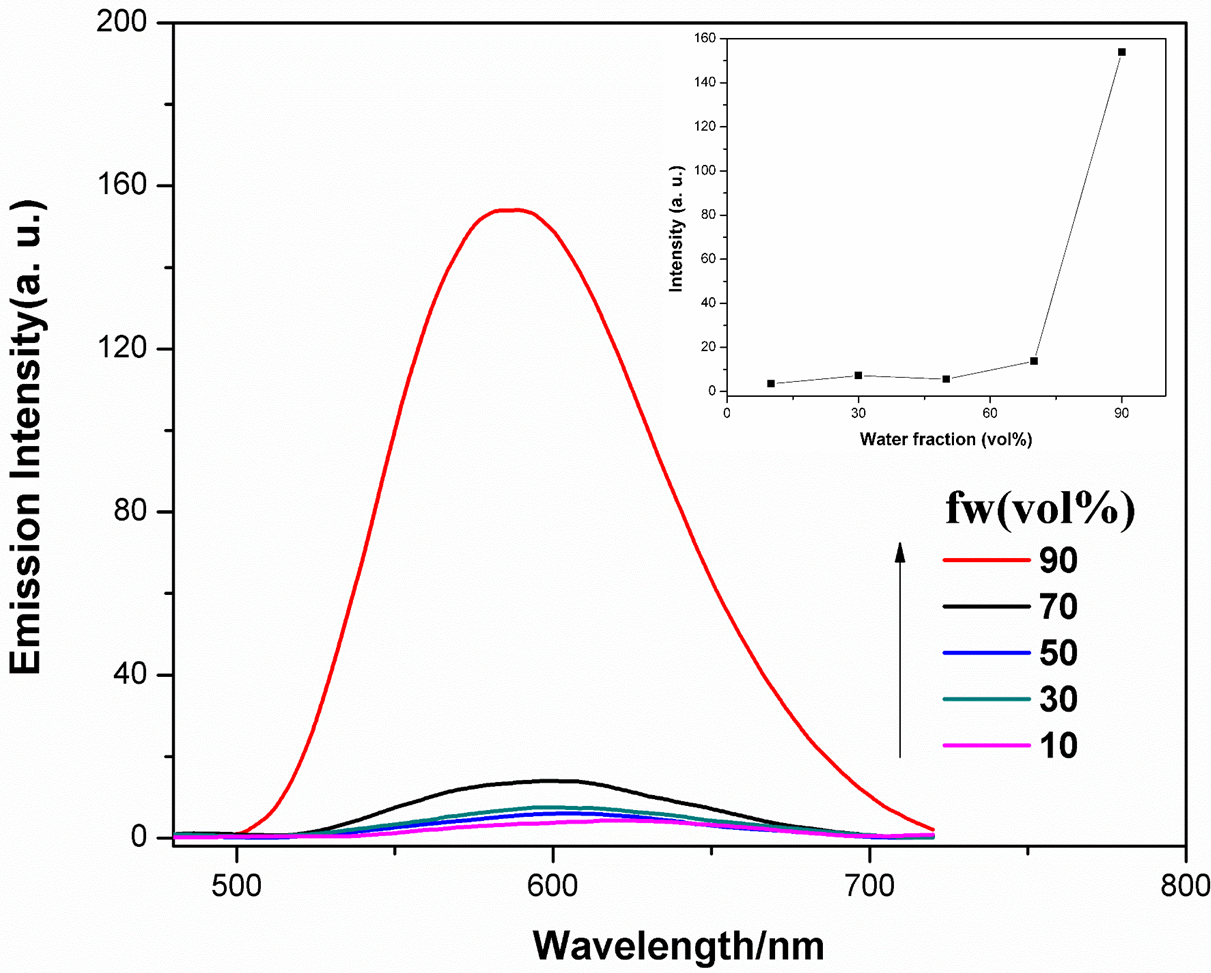


**Figure S15** Emission spectra of **Ir5** in DMSO/PBS mixtures with different water

fractions at 298K with an excitation wavelength of 381 nm.


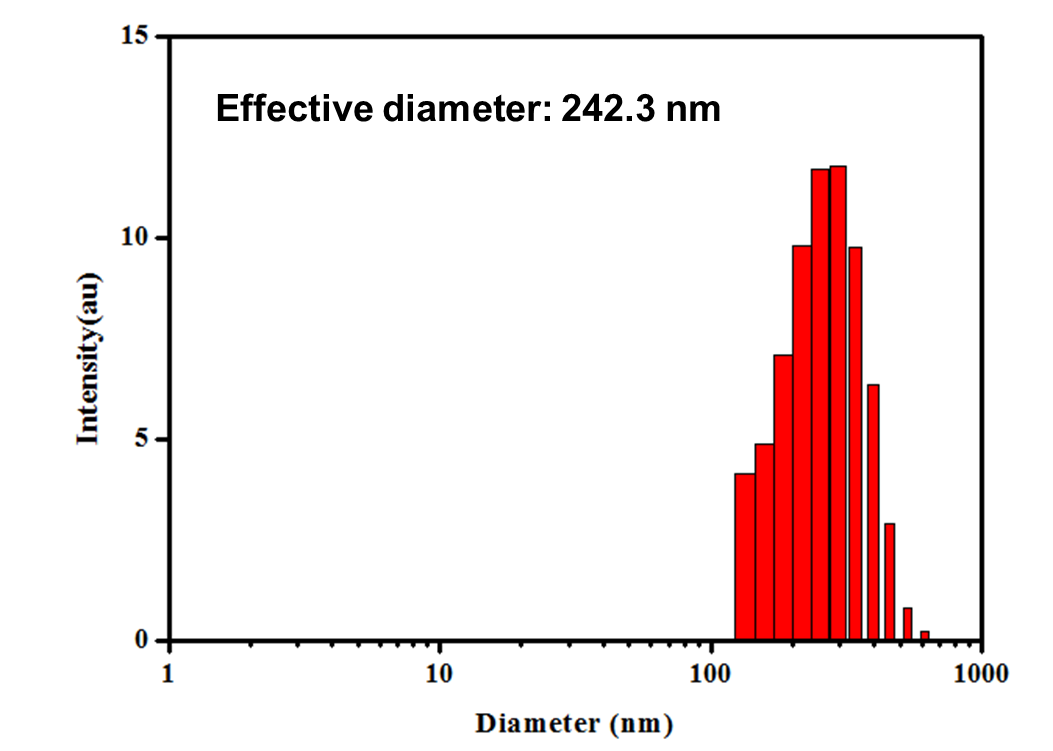


**Figure S16** Size distribution of **Ir1** aggregates in DMSO/PBS mixtures with 90% water fraction. Concentration: 10 μM.


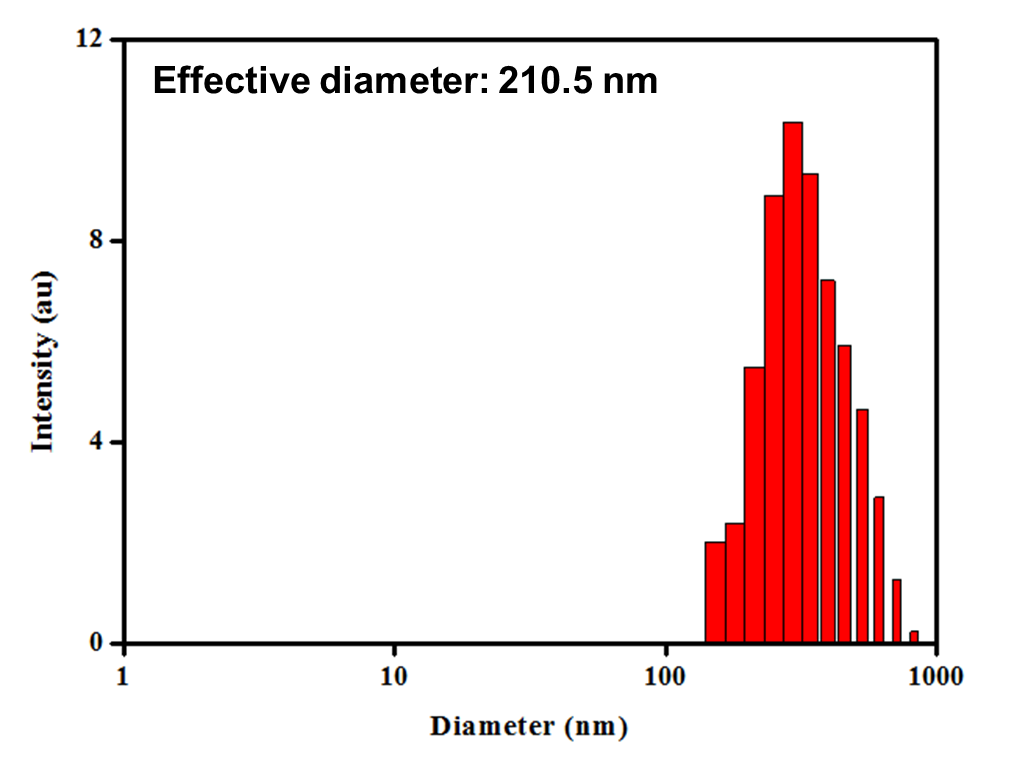


**Figure S17** Size distribution of **Ir2** aggregates in DMSO/PBS mixtures with 90% water fraction. Concentration: 10 μM.


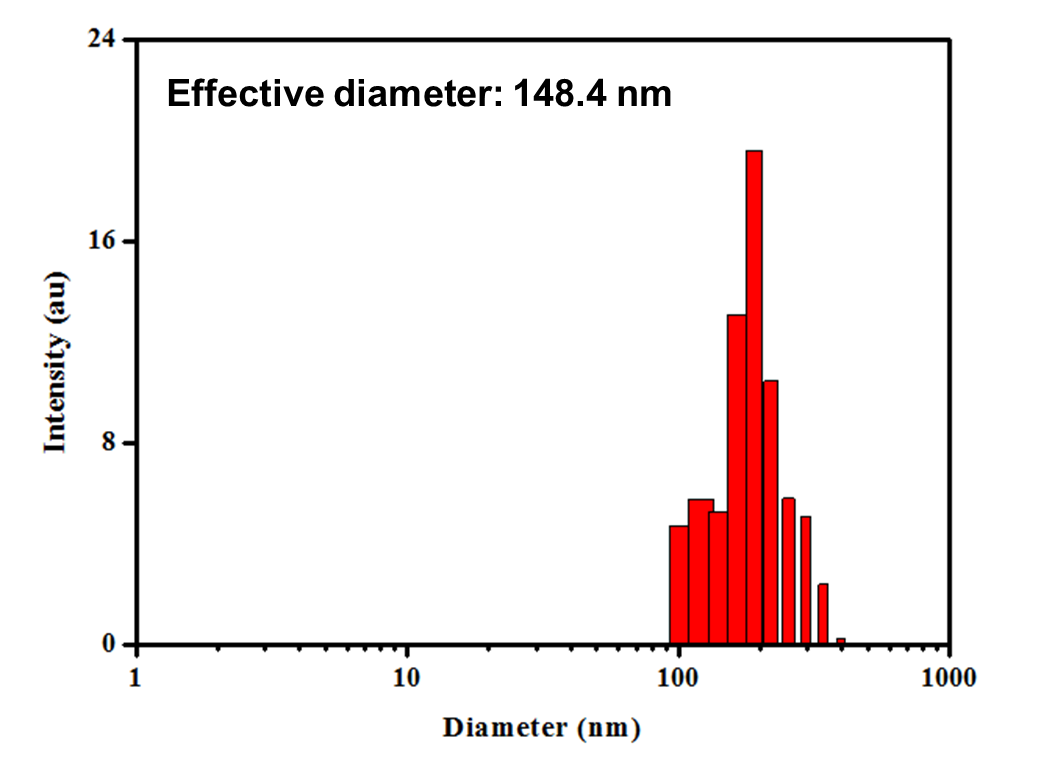


**Figure S18** Size distribution of **Ir3** aggregates in DMSO/PBS mixtures with 90% water fraction. Concentration: 10 μM.


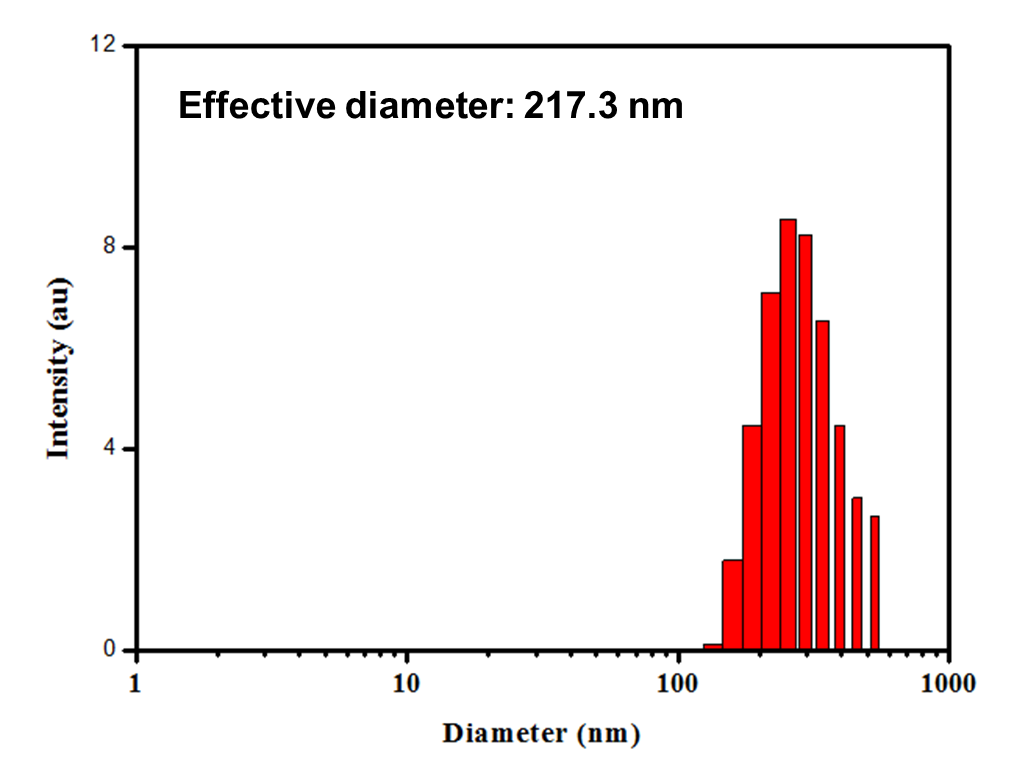


**Figure S19** Size distribution of **Ir4** aggregates in DMSO/PBS mixtures with 90% water fraction. Concentration: 10 μM.


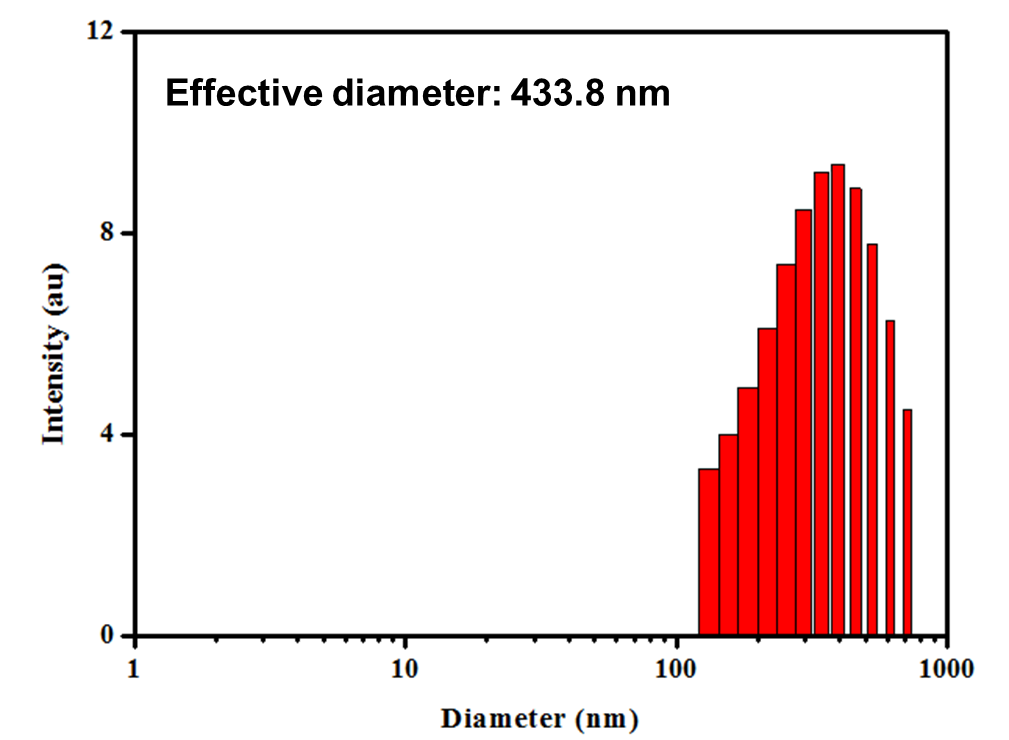


**Figure S20** Size distribution of **Ir5** aggregates in DMSO/PBS mixtures with 90% water fraction. Concentration: 10 μM.


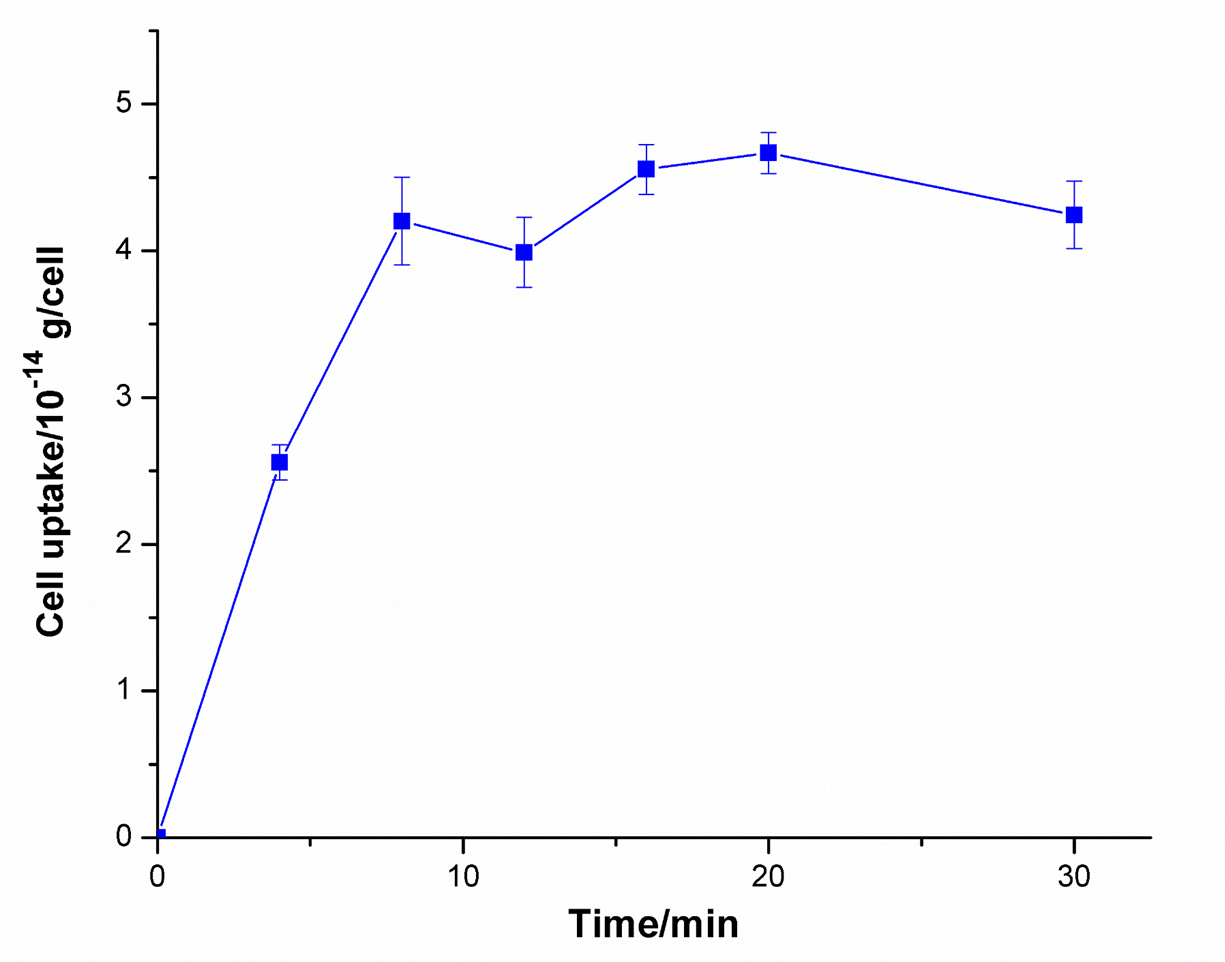


**Figure S21** Time-dependent cell uptake of **Ir1** by ICP-MS.


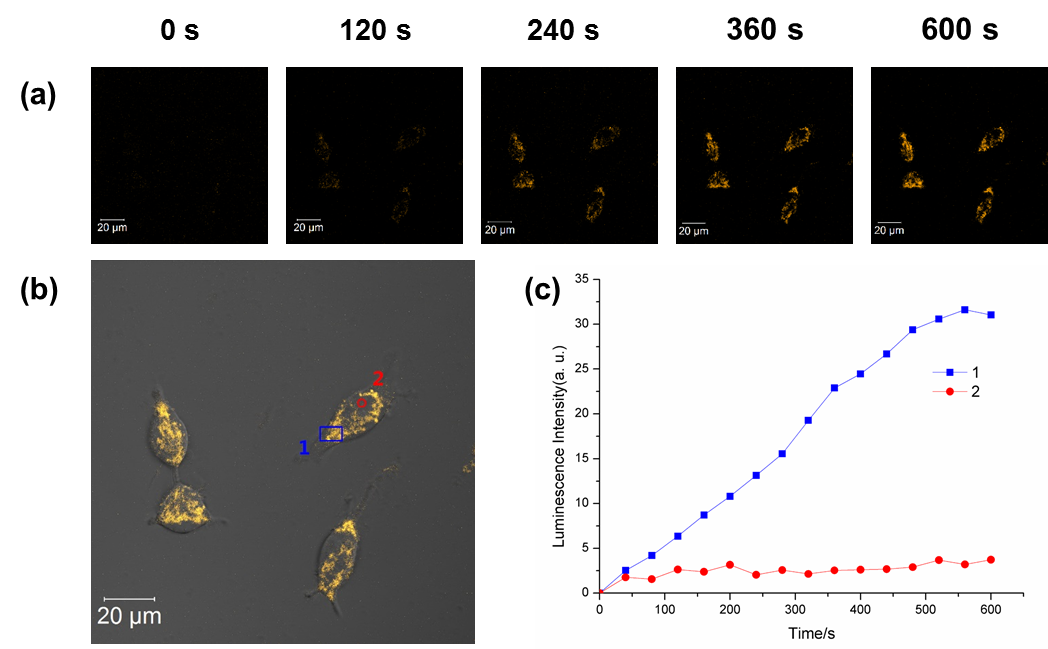


**Figure S22** Real-time monitoring of staining with **Ir1**. (a) Luminescenc in different time (b) Images of living HeLa cells incubated with 200 nM **Ir1** in DMSO/PBS (pH 7.4, 1:50, v/v) at 37 °C at selected time points (λ_ex_ = 405 nm, λ_em_ = 590 ± 30 nm; (c) Time course of luminescence intensity in the whole cell (region 1) and nucleus (region 2)


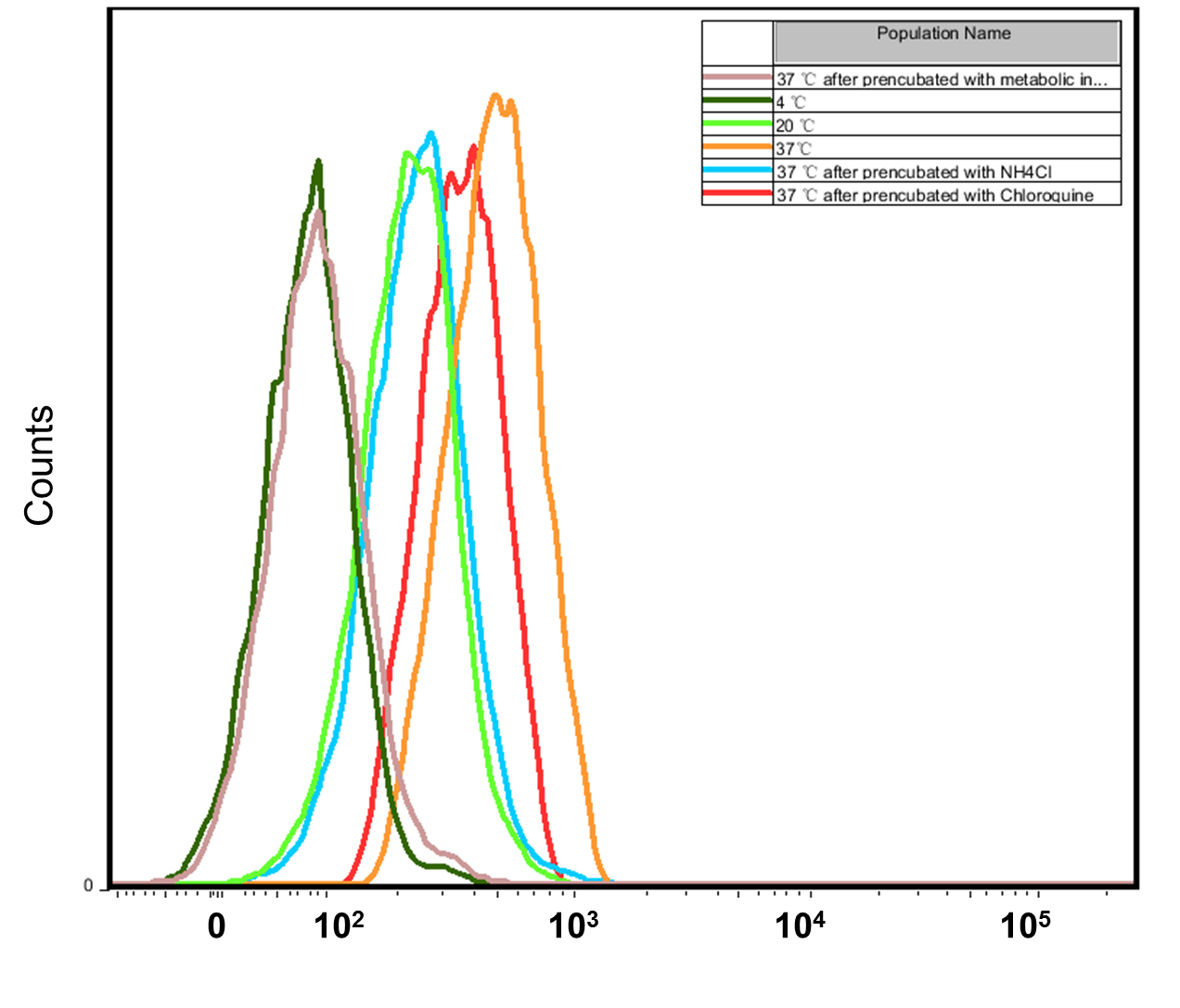


**Figure S23** Flow cytometric histogram profile of cellular uptake of **Ir1** in HeLa cells. HeLa cells were incubated with 500 nM **Ir1** for 8 min at 37 °C (orange), 20°C (light green), 4 °C (dark green), and 37 °C after the cells were preincubated with metabolic inhibitors 2-deoxy-D-glucose (50 mM) and oligomycin (5 μM) in PBS for 1 h at 37°C (light purple), endocytic inhibitors NH_4_Cl (50 mM) (azure) and chloroquine (50 μM) (red) in PBS for 1 h at 37°C, respectively.


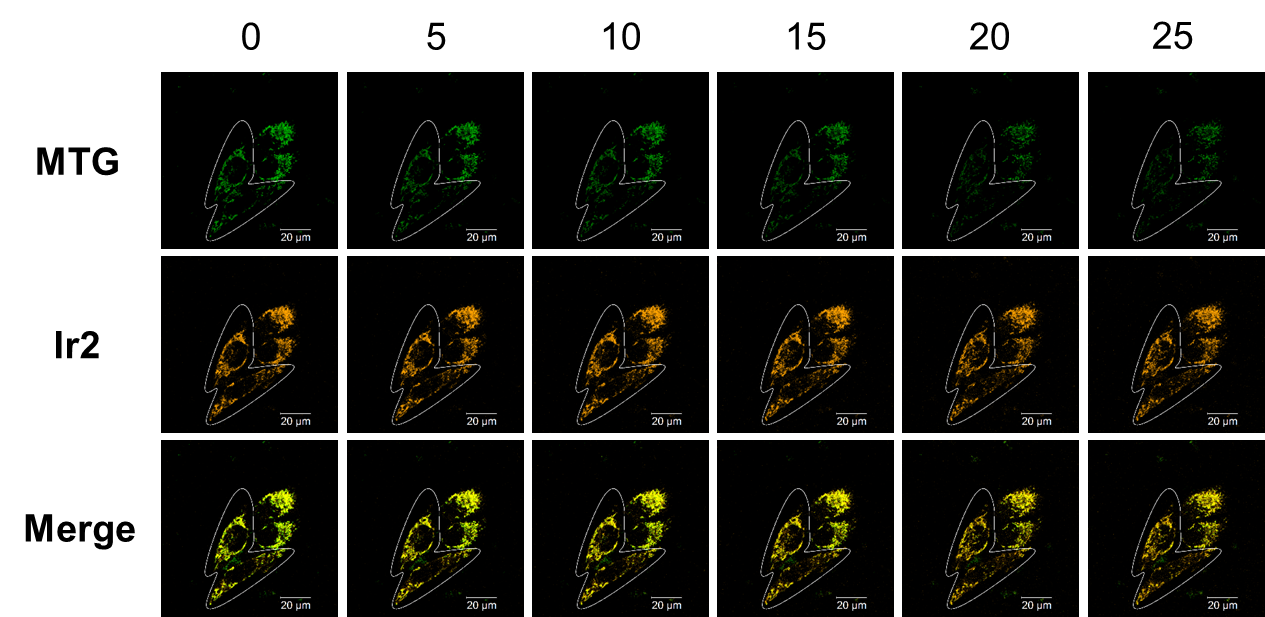


**Figure S24** Photobleaching experiments of **Ir2** in HeLa cells.


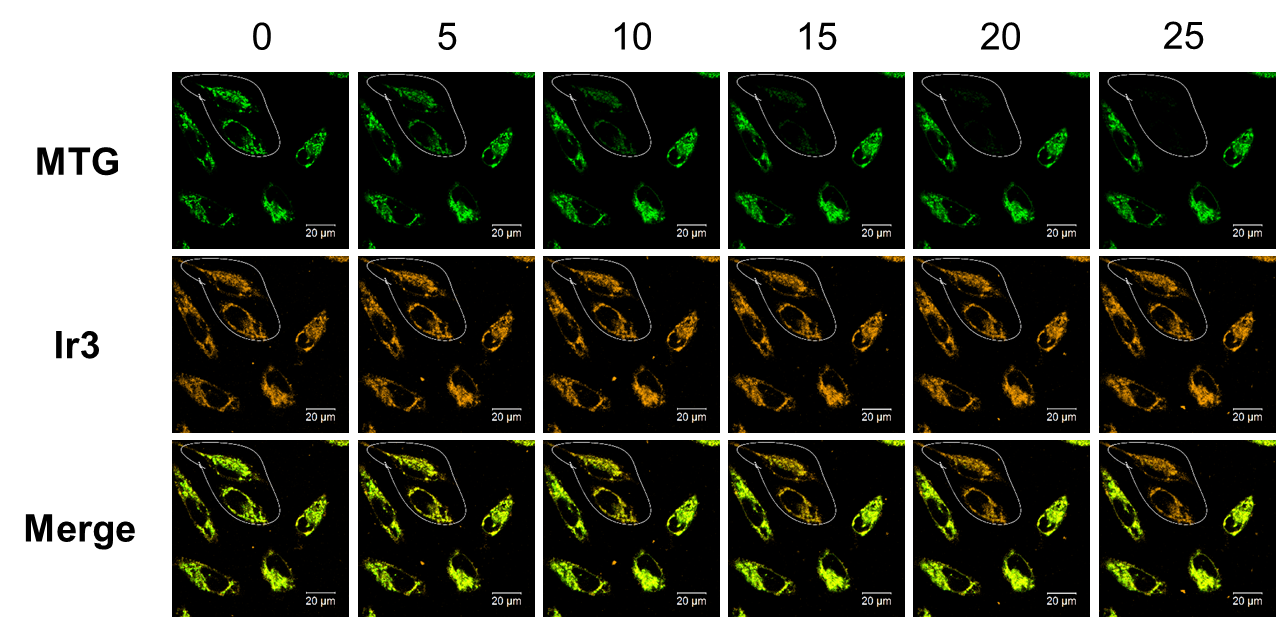


**Figure S25** Photobleaching experiments of **Ir3** in HeLa cells


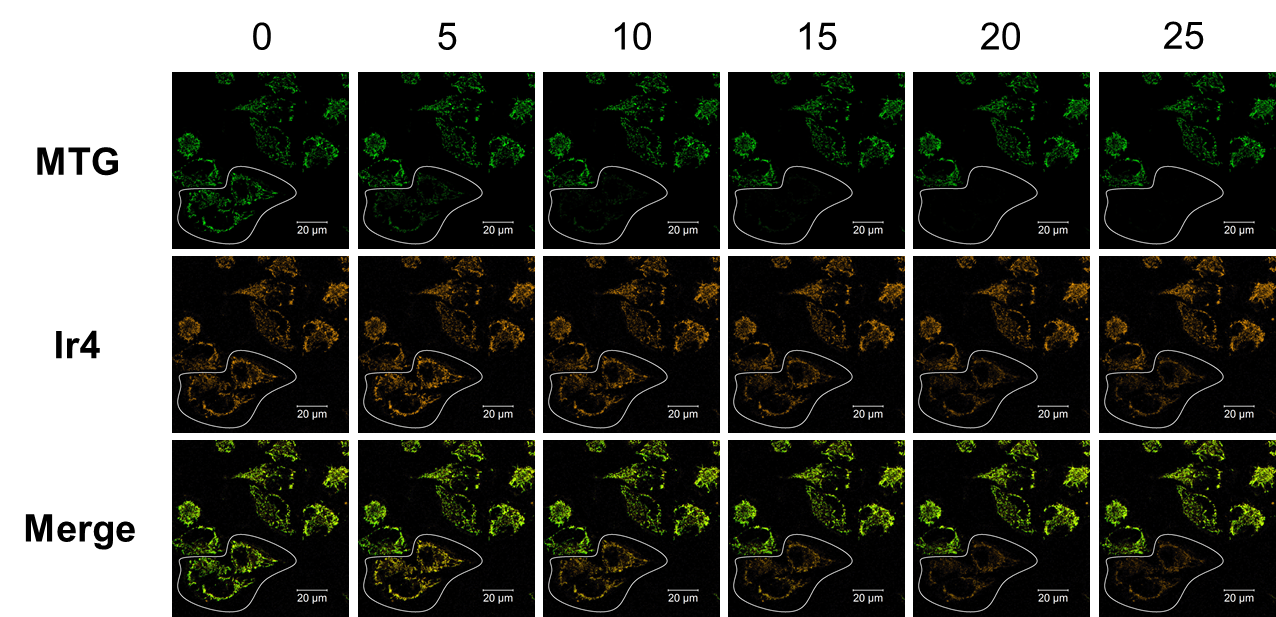


**Figure S26** Photobleaching experiments of **Ir4** in HeLa cells


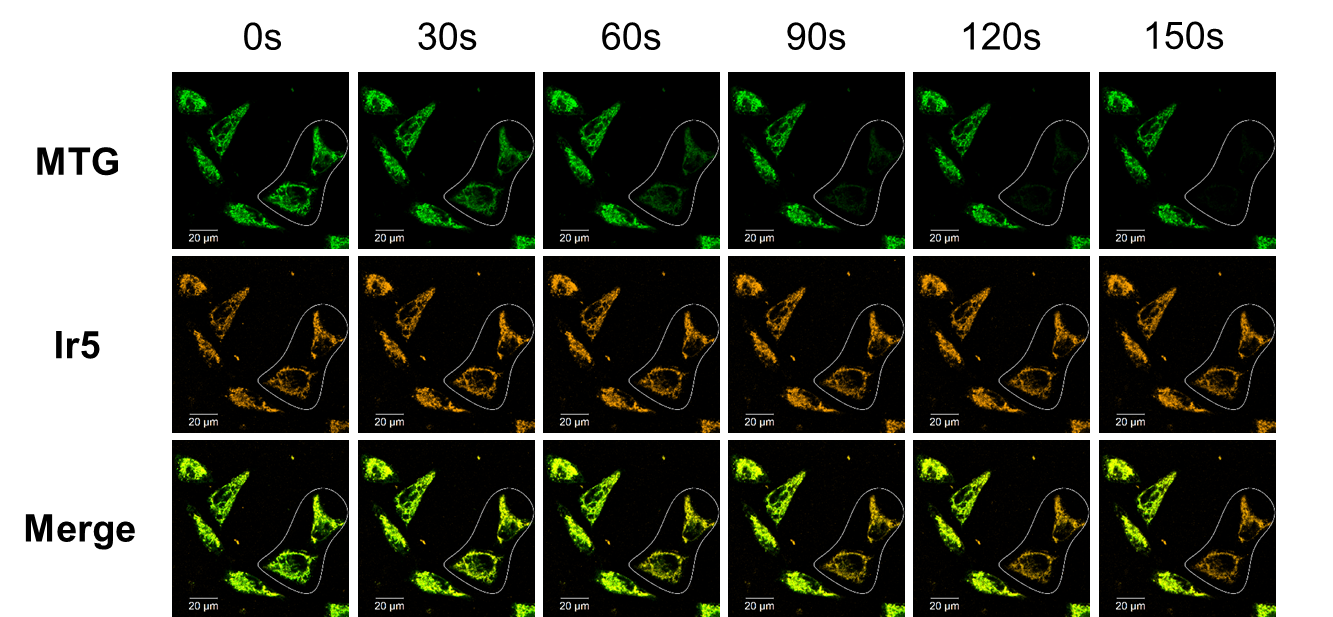


**Figure S27** Photobleaching experiments of **Ir5** in HeLa cells.


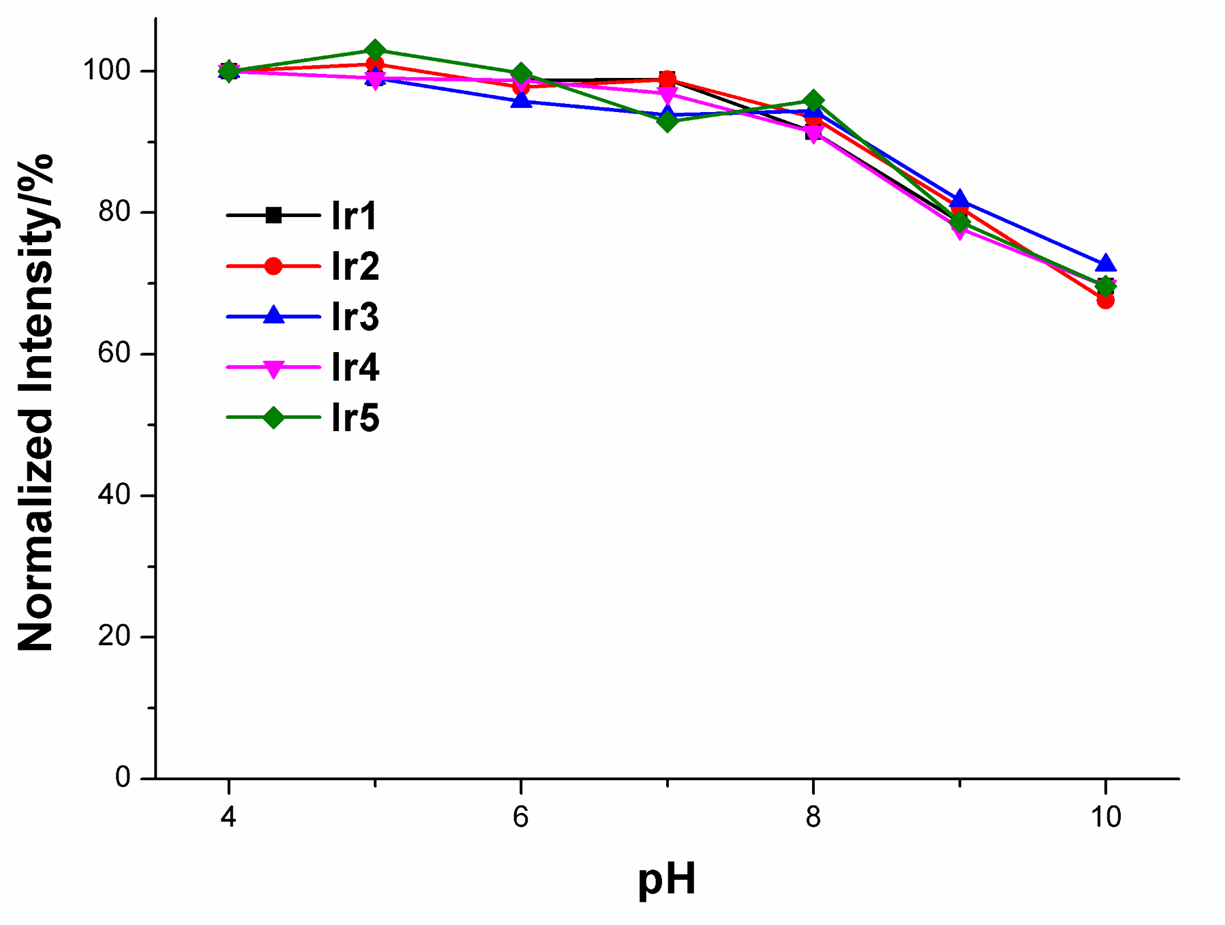


**Figure S28** Emission intensity of 10 μM **Ir1-Ir5** at 590 nm under different pH in a Britton-Robinson buffer.


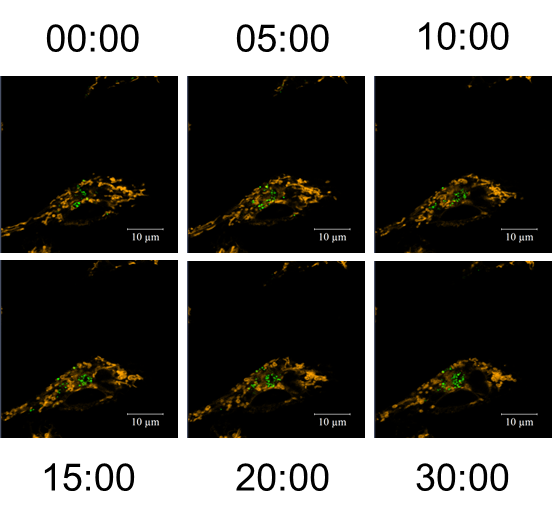


**Figure S29** Confocal images of HeLa cells stained with **Ir1** (500 nM, orange) and LTG (100 nM, green) without CCCP. All the images share the same scale bar: 10 μm.

**Table S1** Photophysical data of **Ir1-Ir5** in DMSO/PBS(v/v=1:9).

| Complex | λ _abs_/nm | λ_em_/nm | τ/ns |
| --- | --- | --- | --- |
| **Ir1** | 381 | 596 | 130.2 |
| **Ir2** | 380 | 592 | 121.5 |
| **Ir3** | 382 | 593 | 115.9 |
| **Ir4** | 380 | 591 | 134.3 |
| **Ir5** | 384 | 592 | 127.6 |

**Videos**

**Video S1.** Real-Time Monitoring of Mitophagy in HeLa cells stained with **Ir1** (500 nM, orange) and LTG (100 nM, green) at 37 ºC. Cell imaging was then captured with Zeiss LSM 710 NLO confocal microscope (63×/NA 1.4 oil immersion objective). Emission was collected at 590 ± 30 nm upon excitation at 405 nm.
